# Identification of novel gene signature for lung adenocarcinoma by machine learning to predict immunotherapy and prognosis

**DOI:** 10.3389/fimmu.2023.1177847

**Published:** 2023-07-31

**Authors:** Jianfeng Shu, Jinni Jiang, Guofang Zhao

**Affiliations:** ^1^ Department of Thoracic Surgery, Ningbo No.2 Hospital, Ningbo, China; ^2^ Ningbo Institute of Life and Health Industry, University of Chinese Academy of Sciences, Ningbo, China

**Keywords:** lung adenocarcinoma, single-cell analysis, immune cells, molecular subtyping, risk model, immunotherapy

## Abstract

**Background:**

Lung adenocarcinoma (LUAD) as a frequent type of lung cancer has a 5-year overall survival rate of lower than 20% among patients with advanced lung cancer. This study aims to construct a risk model to guide immunotherapy in LUAD patients effectively.

**Materials and methods:**

LUAD Bulk RNA-seq data for the construction of a model, single-cell RNA sequencing (scRNA-seq) data (GSE203360) for cell cluster analysis, and microarray data (GSE31210) for validation were collected from The Cancer Genome Atlas (TCGA) and Gene Expression Omnibus (GEO) database. We used the Seurat R package to filter and process scRNA-seq data. Sample clustering was performed in the ConsensusClusterPlus R package. Differentially expressed genes (DEGs) between two groups were mined by the Limma R package. MCP-counter, CIBERSORT, ssGSEA, and ESTIMATE were employed to evaluate immune characteristics. Stepwise multivariate analysis, Univariate Cox analysis, and Lasso regression analysis were conducted to identify key prognostic genes and were used to construct the risk model. Key prognostic gene expressions were explored by RT-qPCR and Western blot assay.

**Results:**

A total of 27 immune cell marker genes associated with prognosis were identified for subtyping LUAD samples into clusters C3, C2, and C1. C1 had the longest overall survival and highest immune infiltration among them, followed by C2 and C3. Oncogenic pathways such as VEGF, EFGR, and MAPK were more activated in C3 compared to the other two clusters. Based on the DEGs among clusters, we confirmed seven key prognostic genes including CPA3, S100P, PTTG1, LOXL2, MELTF, PKP2, and TMPRSS11E. Two risk groups defined by the seven-gene risk model presented distinct responses to immunotherapy and chemotherapy, immune infiltration, and prognosis. The mRNA and protein level of CPA3 was decreased, while the remaining six gene levels were increased in clinical tumor tissues.

**Conclusion:**

Immune cell markers are effective in clustering LUAD samples into different subtypes, and they play important roles in regulating the immune microenvironment and cancer development. In addition, the seven-gene risk model may serve as a guide for assisting in personalized treatment in LUAD patients.

## Introduction

Lung cancer has the largest proportion among all cancer types (1). Lung adenocarcinoma (LUAD) accounts for over half of all lung cancer patients. The development of diagnosis and treatment improves the overall survival time and survival quality of LUAD patients. However, the 5-year survival of metastatic LUAD patients is lower than 20% (2). Worse still, the incidence and mortality of lung cancer are rising (3). Therefore, in order to identify new therapeutic targets and improve patient survival, there is an urgent need to further develop specific prognostic prediction methods for LUAD patients.

For these metastatic cancer patients, targeted therapy such as EGFR and ALK inhibitors have been developed, but they only show favorable efficiency to specific patients with EGFR and ALK mutations (4, 5). A monumental breakthrough in cancer treatment and immunotherapy has revolutionized the field of oncology by harnessing the body’s natural defenses to eliminate malignant cells (6). For example, immunotherapy with immune checkpoint inhibitors such as programmed cell death ligand 1 (PD-L1) and programmed cell death protein 1 (PD-1) inhibitors has shown satisfying outcomes in multiple cancer types including lung cancer (7). Nevertheless, the response to anti-PD-1/PD-L1 therapy in metastatic cancer patients is unfavorable. Compared to chemotherapy and radiotherapy, immunotherapy has more benefits in treating cancer patients at the late stages, such as low side effects, low toxicity, and low resistance. Therefore, it is critical to improve the efficiency of immunotherapy.

Previous studies have shown that the response to immunotherapy is greatly affected by immune microenvironment and tumor heterogeneity (8, 9). In this study, we deciphered scRNA-seq data and the immune landscape of LUAD patients. In combination with bulk RNA-seq data, three molecular subtypes showing disparate prognosis, immune characteristics, and genomic features were categorized. In addition, a seven-gene risk model capable of dividing high-risk and low-risk LUAD patients was established. Two risk groups (high risk and low risk) manifested differential responses to immunotherapy and chemotherapy, prognosis, and immune microenvironment.

## Materials and methods

### Data collection of LUAD samples

From the Gene Expression Omnibus (GEO) database, scRNA-seq data (GSE203360) and microarray expression profiles (GSE31210) of LUAD samples were accessed (10–12). From the Cancer Genome Atlas (TCGA) database, Bulk RNA-seq data (transcripts per million, TPM) of LUAD samples were obtained on the Sangerbox platform (13, 14).

### Preprocessing microarray and bulk RNA-seq data

For microarray data, we annotated probes into gene symbols according to the GPL570 platform. We excluded the probes that matched multiple genes. When one gene had multiple probes, the average expression was used. Totally, 246 LUAD samples in the GSE31210 dataset were included. For bulk RNA-seq data (named as TCGA dataset), the LUAD samples with a survival time > 0 and survival information were retained. Ensembl ID was transferred to the gene symbol. Protein-coding genes were included. Totally, 500 LUAD and 59 normal samples in the TCGA dataset were included.

### Processing of scRNA-seq data

GSE203360 dataset includes scRNA-seq data of six LUAD samples. Quality control procedure was conducted to filter single cells: 1) Each gene expressed more than 200 genes and was expressed in more than three cells; 2) The quantity of expressed genes in cells was between 100 and 5000; 3) The percentage of mitochondrial genes in one cell was less than 30%; 4) The number of unique molecular identifiers (UMI) was more than 100. Finally, a total of 18694 cells were included under the above filtering.

SEURAT R package (15) was utilized to process the scRNA-seq data. First, scRNA-seq data was log-normalized, and the “FindVariableFeatures” function was performed to discriminate highly variable genes. To eliminate the batch effects of six samples, “FindIntegrationAnchors” and “IntegrateData” functions were used. Then, we scaled the scRNA-seq data using the “ScaleData” function and implemented PCA to decrease dimensionality. Subsequently, “FindClusters” functions and “FindNeighbors” were conducted to cluster single cells into different subgroups under conditions of dim = 40 and resolution = 0.2. Finally, single cells were visualized by t-SNE plot through the “RunTSNE” function.

Next, we obtained cell markers of different cell types from CellMarker2.0 (16) and annotated single cells based on these markers. The “FindAllMarkers” function was used to filter DEGs of different cell types under the criteria of logFC = 0.5 and Minpct=0.5. DEGs with P < 0.05 were determined as marker genes of cell types. Only immune cells were remaining for further analysis. The WebGestaltR package (17) was employed to assess KEGG pathways and GO terms of marker genes.

### Identification of molecular subtypes

First, DEGs were screened with the limma R package by comparing LUAD samples to normal samples in the TCGA dataset (18). DEGs with false discovery rates (FDR) < 0.05 and |log2FC| > 1 were retained. Univariate Cox regression analysis was performed on marker genes of immune cells screened from single-cell analysis. The marker genes with P < 0.01 were included for further analysis. Then, the intersection of DEGs and marker genes was selected as a basis for molecular subtyping.

Based on the expression profiles of intersected genes, the ConsensusClusterPlus R package (19) carried out unsupervised consensus clustering. A total of 500 bootstraps were conducted using the “hc” algorithm and “Pearson” distance. Each bootstrap contained 80% of TCGA samples. Cluster number k from two to 10 was set, and the optimal k could be determined based on the cumulative distribution function (CDF) and ConsensusClusterPlus method. The same procedure was performed in the GSE31210 dataset.

### Immune characteristics and pathway analysis

We used multiple methodologies to estimate immune characteristics. CIBERSORT algorithm calculated the enrichment score for 22 kinds of immune cells (20). Stromal infiltration and immune infiltration were evaluated by ESTIMATE (21). We obtained gene signatures of 28 immune cells from previous studies and analyzed their enrichment scores using ssGSEA (22, 23). Moreover, Microenvironment Cell Populations-counter (MCP-counter) was employed to assess stromal and immune cell infiltration (24). The enrichment of tumor-related pathways was assessed by the PROGENy (Pathway RespOnsive GENes) algorithm (25).

### Establishment of a risk model

First of all, DEGs were distinguished from different clusters [FDR < 0.05 and |log2FC| > log2(1.5)]. We selected the intersected DEGs of different clusters for univariate Cox regression analysis, and the DEGs with P < 0.05 were screened. Then, Lasso regression compressed gene numbers in the glmnet R package (26). With the increase in the lambda value, the coefficients of genes were close to zero. A model was built by 10-fold cross-validation, and the optimal one was chosen according to the lambda value. Stepwise multivariate analysis (stepAIC) was further performed in the Mass R package to reduce the gene number (27). To reach the optimal model, the variables were deleted accordingly to reduce the AIC value. A sufficient fitting degree with fewer variables was ensured by StepAIC. The risk model was finally determined as risk score = Σ(exp i* beta i), where beta represents multivariate coefficients, exp represents expression levels, and i represents genes.

### Evaluation of the risk model

According to the formula of the risk model, we calculated the risk score for each sample and divided them into groups of high and low risks using the median risk score. Kaplan-Meier survival analysis showed the prognosis difference between the two risk groups. Receiver operation characteristic (ROC) curve analysis evaluated the survival prediction efficiency (1-year, 3-year, and 5-year) of the risk model. A nomogram was developed combining the model with clinical features.

### The risk model to predict chemotherapy and immunotherapy response

Tumor Immune Dysfunction and Exclusion (TIDE) (http://tide.dfci.harvard.edu/) predicted the response to immune checkpoint blockade (ICB) therapy. The estimated IC50 for assessing the response to eight chemotherapeutic drugs (Erlotinib, Rapamycin, PHA-665752, Roscovitine, Cisplatin, Paclitaxel, BI-2536, and Pyrimethamine) was calculated by pRRophetic R package. IMvigor210 dataset (28) containing the expression data of urothelial carcinoma patients treated by anti-programmed death-ligand 1 (PD-L1) therapy was used to further validate the performance of the risk model. IMvigor210 dataset contains 348 patients divided into complete response (CR), progressive disease (PD), partial response (PR), and stable disease (SD) groups.

### Clinical samples

In this research, we collected five pairs of LUAD and adjacent normal lung tissues from Ningbo No.2 Hospital. Informed consent was obtained from all participants. The study was approved by the Ethics Committee of Ningbo No.2 Hospital.

### RNA preparation and real-time quantitative PCR (RT-qPCR) analysis

TRIzol reagent (Invitrogen, CA, USA) was applied to extract RNA from tissues. PrimeScript™ RT Master Mix (Takara, Dalian, China) was used to synthesize cDNAs to explore genes’ mRNA expression detection according to the manufacturer’s instructions. The RT-qPCR was conducted using a TB Green Premix Ex Taq™ II kit (Takara, Dalian, China) with a 7500 Real-Time PCR System (Applied Biosystems, Foster City, CA) according to construction sequence. The relative expression of genes was performed using a 2^-ΔΔct^ method using an internal reference gene, GAPDH. The primers of genes are shown in **Table 1**.

**Table 1:** The primers of genes.

Gene	Forward primer sequence (5’-3’)	Reverse primer sequence (5’-3’)
CPA3	ATTCACGCACGAGAATGGGT	CCACATGCGGTTCTTTGTCC
S100P	GAGACAGCCATGGGCATGAT	CGTCCAGGTCCTTGAGCAAT
PTTG1	CCAAGGGACCCCTCAAACAA	GGCATCATCTGAGGCAGGAA
LOXL2	CCAGTGTGGTCTGCAGAGAG	CCTGTGCACTGGATCTCGTT
MELTF	GGCACACAACCGTCTTTGAC	GGGGCACAGCAGTTCATAGT
PKP2	GAGATGACTCTGGAGCGAGC	AAGCTTGAGGATGCCACGAA
TMPRSS11E	CCTGATTGTCCTGGCAGTGT	CCTCTCTGCCAAACTCAGCA
GAPDH	AGGGCATCCTGGGCTACAC	GCCAAATTCGTTGTCATACCAG

### Western blot assay

Proteins were extracted in RIPA lysis buffer (P0013B, Beyotime) and the concentration was determined using a BCA Protein assay kit (P0010S, Beyotime). Proteins were separated on sodium dodecyl sulfate-polyacrylamide gels (SDS-PAGE) and then transferred to polyvinylidene fluoride (PVDF) membranes. The membranes were blocked with 5% non-fat dry milk and then incubated overnight with primary antibodies, including anti-CAP3 (1:1000, ARG58412, and Arigo Biolaboratories Corp.), anti-S100P (1:1000, 7677S, and CST), anti-PTTG1 (1:1000, 13445, and CST), anti-LOXL2 (1:1000, ab96233, and Abcam), anti-MELTF (1:1000, 10428-1-AP, and Proteintech), anti-PKP2 (1:1000, ab189323, and Abcam), anti-TMPRSS11E (1:1000, PA5-48775, and Invitrogen Antibodies), and anti-β-actin (1:10000, A5441, and Sigma). Horseradish peroxidase (HRP)-conjugated goat anti-mouse/rabbit IgG secondary antibody was used to incubate membranes at room temperature. The blots were detected by chemiluminescence and imaged on an AlphaView analysis system (ProteinSimle, USA). The quantification of individual protein bands was assessed by densitometry using ImageJ software.

### Statistical analysis

All statistical analysis was done with the R program (v4.2.0). The Wilcoxon test evaluated the two-group difference. Differences among the three groups were analyzed by the Kruskal-Wallis test. Cox analysis and survival analysis both used the log-rank test.

## Results

### Annotating cell types in single-cell data of LUAD patients

**Figure 1:**
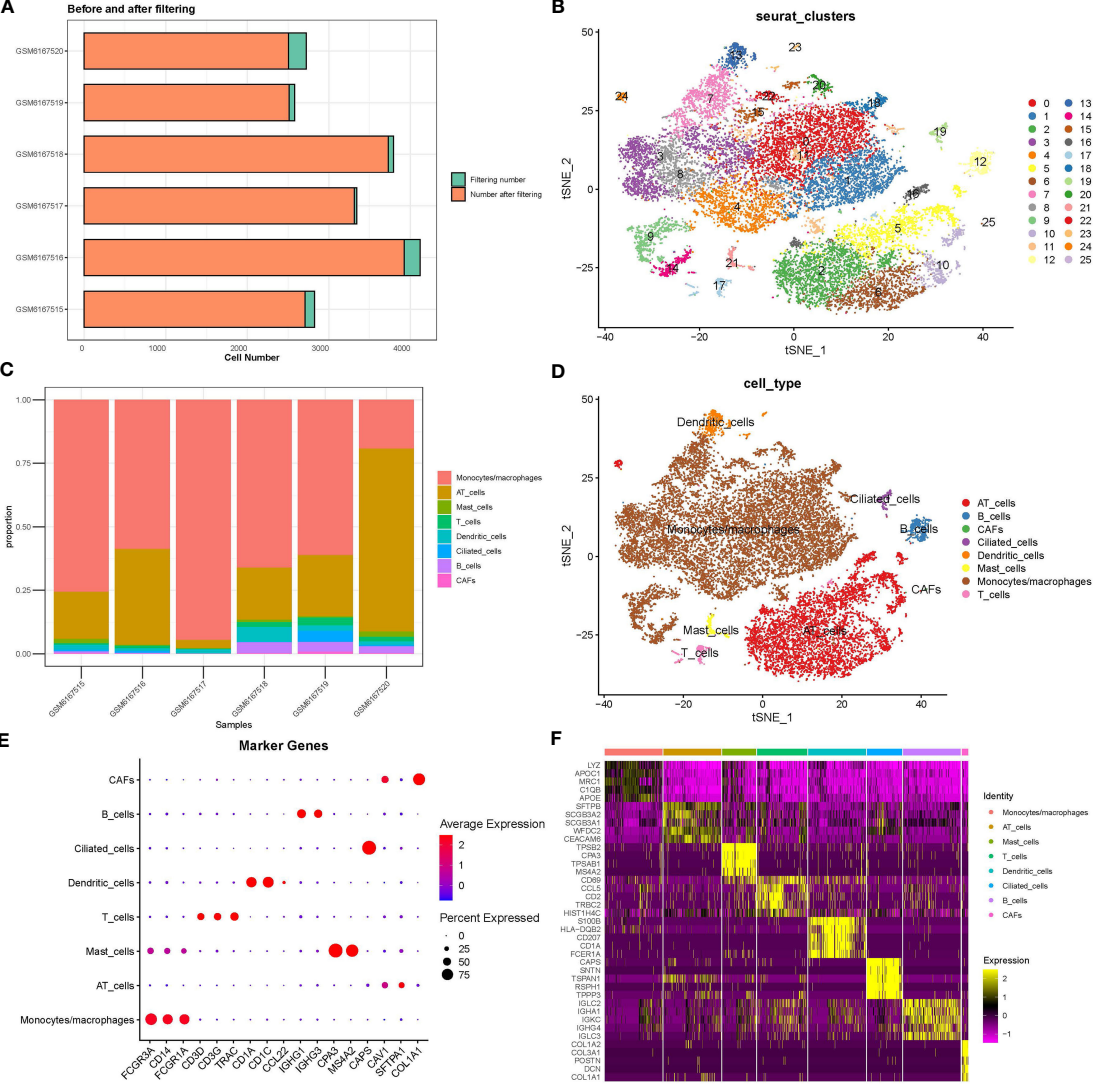
Analysis of scRNA-seq data. **(A)** Cell counts before and after data filtering. **(B)** T-SNE plot of clustering cells. **(C)** The proportion of eight cell types in six samples. **(D)** The distribution of eight cell types is shown in the T-SNE plot. **(E)** The expression of marker genes of eight cell types. **(F)** The top five DEGs (marker genes) of eight cell types. CAF, cancer-associated fibroblasts; AT, alveolar type.

**Figure 2:**
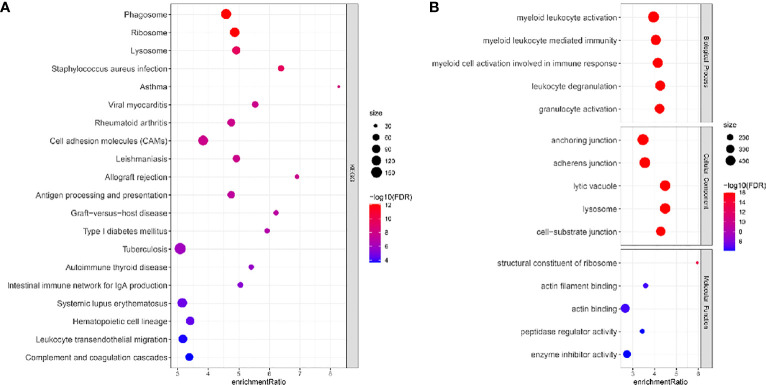
KEGG **(A)** and GO **(B)** function analysis of DEGs of immune cells (monocytes/macrophages, mast cells, T cells, dendritic cells, and B cells) in the TCGA dataset. FDR, false discovery rate.

Single cells were filtered using the Seurat R package and 18694 cells were remaining (**Figures S1A, B** and **Figure 1A**, see the filtering details in Materials and Methods). Then, the expression profiles of single cells were log-normalized and the batch effects of six samples were eliminated (**Figures S1C, D**). Gene expression levels were scaled and single cells were divided into 26 clusters (**Figure 1B**). Further, we annotated these clusters into eight cell types according to the markers from CellMarker2.0 (16) where monocytes/macrophages and AT cells (AT1 (alveolar type I cells) and AT2 (alveolar type II cells)) account for the majority of cells (**Figures 1C–E**; **Table 2**). By assessing the difference in expression profiles among different cell types, we identified DEGs in each cell type and the top five DEGs were visualized (**Figure 1F**). Only the DEGs of five immune cells (dendritic cells, B cells, monocytes/macrophages, mast cells, and T cells) were retained for the subsequent analysis (**Table S1**). These DEGs were defined as the markers of five immune cells. Functional analysis showed that these markers were apparently enriched in immune-related pathways including phagosome and antigen processing and presentation (**Figure 2A**). Moreover, GO analysis revealed that immune-related biological pathways were significantly accumulated such as myeloid leukocyte, myeloid leukocyte activation, and involved myeloid leukocyte-mediated immunity in immune response (**Figure 2B**).

**Table 2:** Twenty-six clusters annotated by marker genes of eight cell types.

Cell type	marker gene	seurat_clusters
Monocytes/macrophages	FCGR3A, CD14, FCGR1A	0,1,3,4,7,8,9,11,14,15,18,20,22
T_cells	CD3D, CD3G, TRAC	17
Dendritic_cells	CD1A, CD1C, CCL22	13,23
B_cells	IGHG1, IGHG3	12
Mast_cells	CPA3, MS4A2	21
Ciliated_cells	CAPS	19
AT cell	CAV1, SFTPA1	2,5,6,10,16,24
CAFs	COL1A1	25

### Molecular subtyping based on the markers of immune cells

**Figure 3:**
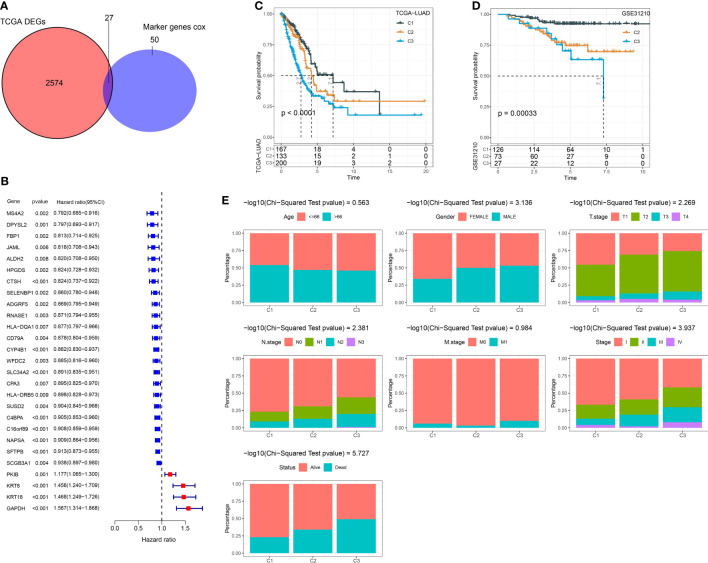
Constructing molecular subtypes based on DEGs. **(A)** Venn plot of DEGs (identified between tumor and normal samples in the TCGA dataset) and prognostic marker genes (identified in scRNA-seq data). **(B)** Univariate Cox regression result of 27 intersected genes. **(C)** Kaplan-Meier survival analysis of three clusters in the TCGA dataset. **(D)** Kaplan-Meier survival analysis of three clusters in the GSE31210 dataset. **(E)** The distribution of different clinical features in three clusters in the TCGA dataset.

We performed univariate Cox regression analysis to ascertain the markers that were significantly associated with LUAD prognosis in the TCGA dataset. A total of 77 marker genes were identified with P < 0.01 (**Table S2**). We further screened dysregulated genes of LUAD by comparing LUAD samples with normal samples in the TCGA dataset. To ensure which markers had a remarkable contribution to LUAD, we selected the intersection between prognostic marker genes and dysregulated genes. Venn plot showed that a total of 27 marker genes were intersected and these genes were used as a basis for the following analysis (**Figures 3A, B**). Next, we conducted consensus clustering based on gene expression profiles of 27 marker genes. According to CDF curves and the area under CDF curves, we confirmed cluster number k = 3 and classified LUAD samples into three clusters (**Figure S2**). Kaplan-Meier survival analysis uncovered that three clusters (C1, C2, and C3) had distinct overall survival in both TCGA (P < 0.0001) and GSE31210 (P = 0.00033) datasets (**Figures 3C, D**). C1 had the longest survival while C3 had the worst survival. The distribution of clinical characteristics in three clusters showed corresponding results with their prognosis. The proportion of patients with late stages was obviously higher than those with early stages in C3 (**Figure 3E**). For example, C3 consisted of higher percentages of stages II to IV than C1 and C2. Not surprisingly, the proportion of dead patients was also significantly higher in C3 compared with that in C1. The results suggested that the subtyping based on the 27 marker genes was reliable.

### Analysis of immune characteristics and biological pathways

**Figure 4:**
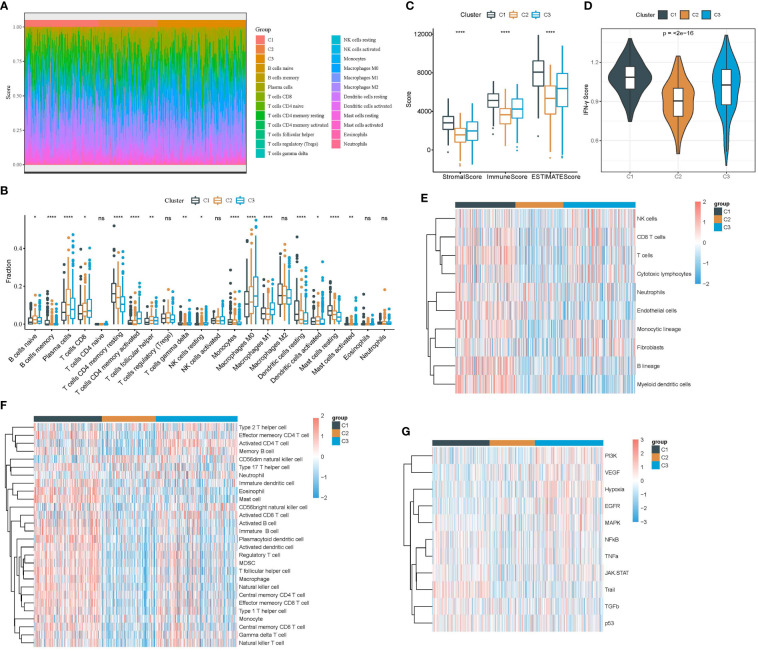
Analysis of immune infiltration and tumor-related pathways in the TCGA dataset. **(A)** The heat map showed the distribution of 22 immune cells in three clusters. **(B)** The box plot showed the estimated enrichment of 22 immune cells in three clusters. **(C)** ESTIMATE analysis revealed immune and stromal infiltration of three clusters. **(D)** IFN-γ expression level in three clusters. **(E)** Comparison of the enrichment of 10 immune-related cells calculated by MCP-counter among three clusters. **(F)** Comparison of the enrichment of 28 immune-related cells calculated by ssGSEA among three clusters. **(G)** Comparison of the enrichment of 11 tumor-related pathways calculated by PROGENy among three clusters. *p < 0.05, **p < 0.01, ****p < 0.0001; ns, no significant.

The tumor microenvironment can indicate an immune response to cancer cells, and a high infiltration of cytotoxic lymphocytes commonly suggests a high activation of anti-cancer response and a relatively favorable outcome. We used several strategies to evaluate the immune infiltration of three clusters in the TCGA dataset. CIBERSORT analysis showed that most immune cells, such as activated memory CD4 T cells, resting memory CD4 T cells, macrophages, and CD8 T cells, were differently enriched in three clusters (**Figures 4A, B**). Compared with C2 and C3, C1 had higher immune infiltration and stromal infiltration (**Figure 4C**). Interferon-γ (IFN-γ) is an essential factor in anti-cancer response and immune regulation. We found that C1 had the highest expression of IFN-γ followed by C3 (**Figure 4D**), indicating that the immune response was relatively active in C1. Moreover, both MCP-counter and ssGSEA revealed the consistent result that most immune cells were more enriched in C1 compared with the other two clusters (**Figures 4E, F**). To figure out the possible molecular mechanisms of different prognoses in three clusters, we evaluated 11 tumor-related pathways using the PROGENy algorithm (25). As a result, most pathways such as PI3K, VEGF, hypoxia, and EGFR were more activated in C3 compared with C1 and C2 (**Figure 4G**). Notably, the pro-apoptotic signaling, Trail, was the most enriched in C1, which may lead to its favorable prognosis.

### Variation characteristics of three clusters

**Figure 5:**
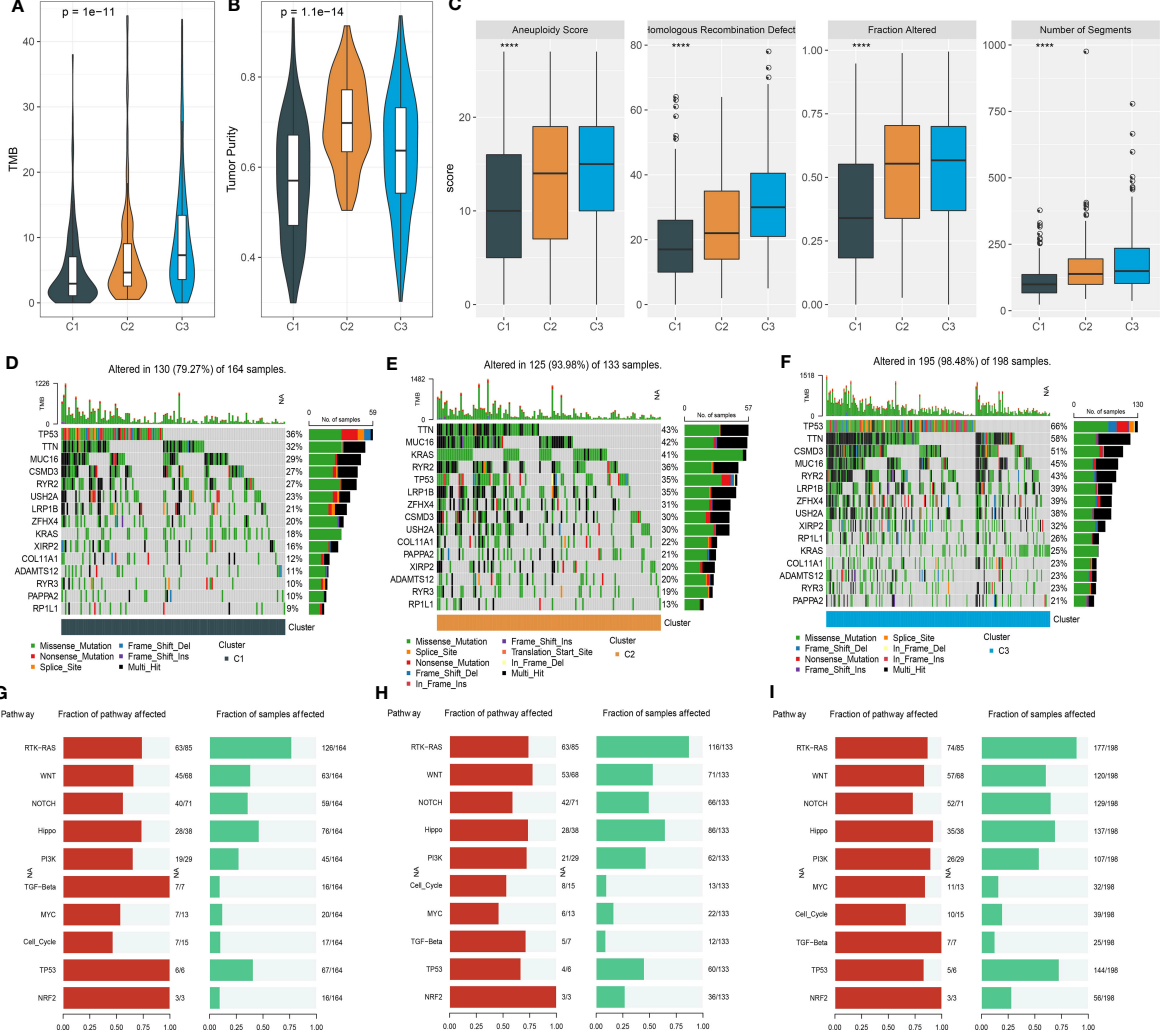
Mutation analysis of three clusters in the TCGA dataset. **(A, B)** TMB and tumor purity of three clusters. **(C)** The scores of aneuploidy, homologous recombination defect, fraction altered, and number of segments. **(D–F)** The top 15 mutated genes in C1 **(D)**, C2 **(E)**, and C3 **(F)**. **(G–I)** The fraction of affected oncogenic pathways and the fraction of samples with mutated pathways in C1 **(G)**, C2 **(H)**, and C3 **(I)**. ****p < 0.0001.

Genomic characteristics have been reported to be linked with cancer development, prognosis, and response to immunotherapy. We obtained several genomic features from previous studies (29, 30). C3 showed the highest tumor mutation burden (TMB) while C1 had the lowest tumor purity (**Figures 5A, B**). In addition, C1 also had the lowest scores of aneuploidy, homologous recombination defect, fraction altered, and number of segments (**Figure 5C**). Furthermore, we assessed the frequencies of gene mutation and screened the significantly mutated genes in three clusters. The top 15 mutated genes of three clusters were listed (**Figures 5D–F**). The mutation frequencies of the top 15 genes varied largely among three clusters. Especially, TP53 had the highest mutation rate in C3 with a mutation frequency of 66%, while the frequencies were 36% and 35% in C1 and C2, respectively. Notably, KRAS presented an extremely higher mutation rate in C2 (41%) compared with C1 (18%) and C3 (25%). To figure out the influence of mutations in oncogenic pathways, we employed maftools (31) to calculate the mutation frequencies of genes of 10 oncogenic pathways. Six of 10 pathways were evidently affected in LUAD samples, including RTK-RAS, WNT, NOTCH, Hippo, PI3K, and TP53 (**Figures 5G–I**). C3 had the largest proportion of affected pathways compared to the other two clusters, indicating that the biological functions of these oncogenic pathways may be largely interfered with.

### Construction of a risk model based on the DEGs in three clusters

Given that the three clusters had different prognoses, immune microenvironments, mutation characteristics, and enrichment of biological pathways, we next attempted to dig out potential key genes contributable to their outcomes. Therefore, we used the limma R package to excavate the DEGs in C1 vs. other, C2 vs. other, and C3 vs. other [FDR < 0.05 and |log2FC| > log2(1.5)]. As a result, we identified 1587 DEGs in C1, 900 DEGs in C2, and 1437 DEGs in C3 (**Figures S3A–C**). Venn plot showed that the DEGs of three groups shared 133 overlapped ones (**Figure S3D**). KEGG analysis showed that the DEGs of C3 were significantly enriched in the cell cycle, p53 signaling pathway, and microRNAs in cancer (**Figure S3E**). The 133 common DEGs were also enriched in the cell cycle (**Figure S3F**), suggesting that these DEGs may have an influence on different prognoses by regulating the cell cycle pathway.

**Figure 6:**
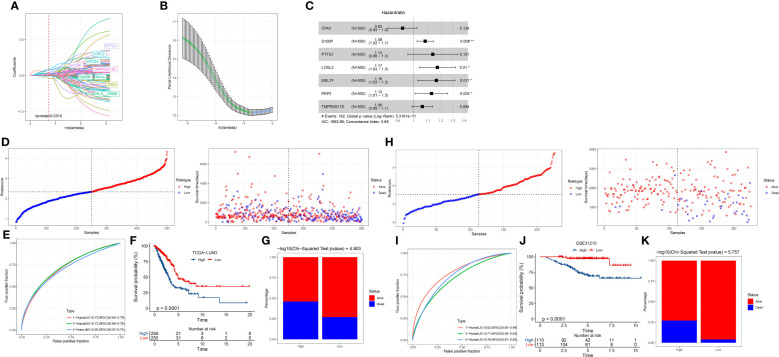
Establishment and verification of a risk model. **(A)** The change of Lasso coefficients with the increasing lambda. **(B)** Partial likelihood of deviance from changing lambda values. **(C)** The hazard ratio of seven prognostic genes was analyzed by stepAIC. **(D)** The division of two risk groups and the distribution of samples ranking by risk score in the TCGA dataset. **(E)** ROC curve of 1-year, 3-year, and 5-year survival in TCGA dataset. **(F)** Kaplan-Meier survival curve of high- and low-risk groups in the TCGA dataset. **(G)** The percentage of alive and dead samples in the two risk groups in the TCGA dataset. **(H–K)** The validation of the risk model in the GSE31210 dataset.

To further explore the potential key DEGs among 133 DEGs, we performed univariate Cox regression analysis and identified 109 DEGs associated with overall survival (**Table S3**). Then, using Lasso regression analysis, we compressed the coefficients of DEGs to retain the important DEGs. The Lasso model reached the optimal when the lambda value = 0.0315, and a total of 15 DEGs were remaining (**Figures 6A, B**). To further reduce the number of genes, stepAIC was employed to ensure a sufficient fitting degree with the least number of genes. As a result, seven key prognostic genes were determined to construct a risk model (**Figure 6C**).

Risk Score = -0.073*CPA3 + 0.077*S100P + 0.128*PTTG1 + 0.158*LOXL2 + 0.152*MELTF + 0.119*PKP2 + 0.058*TMPRSS11E

### Assessment of the seven-gene risk model

We determined the risk score for each tumor sample in the TCGA dataset using the risk model. The median value of the risk score was used to identify the high-risk and low-risk groups (**Figure 6D**). More samples in the high-risk group had a dead status than in the low-risk group (**Figures 6D, G**). With AUCs of 0.72, 0.72, and 0.65, respectively, the ROC curve showed that the risk model was effective for predicting 1-year, 3-year, and 5-year survival (**Figure 6E**). The two risk groups had varied overall survival rates, as indicated by the Kaplan-Meier survival curve (P < 0.0001; **Figure 6F**). We observed consistent results in the GSE31210 dataset (**Figures 6H–K**), demonstrating the robustness and reliability of the seven-gene risk model. In addition, we evaluated the performance of the risk model in the samples with different clinical characteristics. Survival analysis showed that the risk model was effective in distinguishing high-risk and low-risk samples with different clinical features including TNM stage, age, and gender (**Figure S4**).

### Establishing a nomogram to optimize the application of the risk model

**Figure 7:**
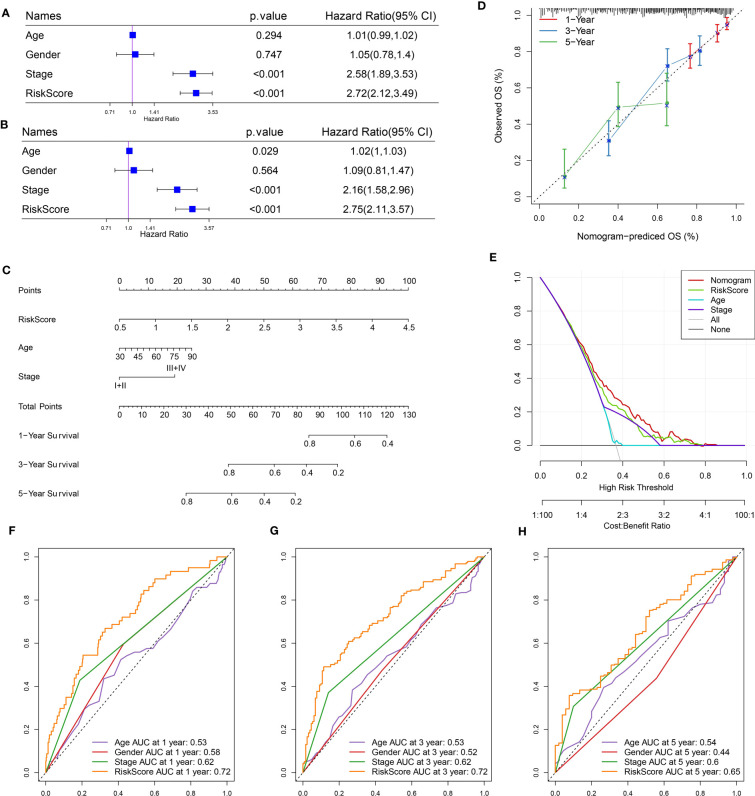
Optimization of the risk model in the TCGA dataset. **(A, B)** Univariate **(A)** and multivariate **(B)** Cox regression analysis of age, gender, stage, and risk score. **(C)** A nomogram based on risk score and stage. **(D)** Comparison of observed overall survival (OS) and nomogram-predicted OS. **(E)** Decision curve analysis of nomogram, stage, and risk score. **(F–H)** ROC curves of age, gender, stage, risk score, and nomogram at 1 year, 3 years, and 5 years.

Risk score and stage were identified as independent risk factors by univariate and multivariate analyses (**Figures 7A, B**). A nomogram based on risk score and stage was therefore created (**Figure 7C**). The risk score showed a more significant effect on the total points compared to the stage. The calibration curve presented that the predicted 1-year, 3-year, and 5-year survival was close to the observed ones (**Figure 7D**). Decision curve analysis validated that patients could obtain more benefits from the nomogram compared to the risk score (**Figure 7E**). Also, compared with clinical characteristics including age, gender, and stage, both risk score and nomogram showed higher AUC of 1-year, 3-year, and 5-year survival (**Figures 7F–H**).

### Assessment of immune characteristics

**Figure 8:**
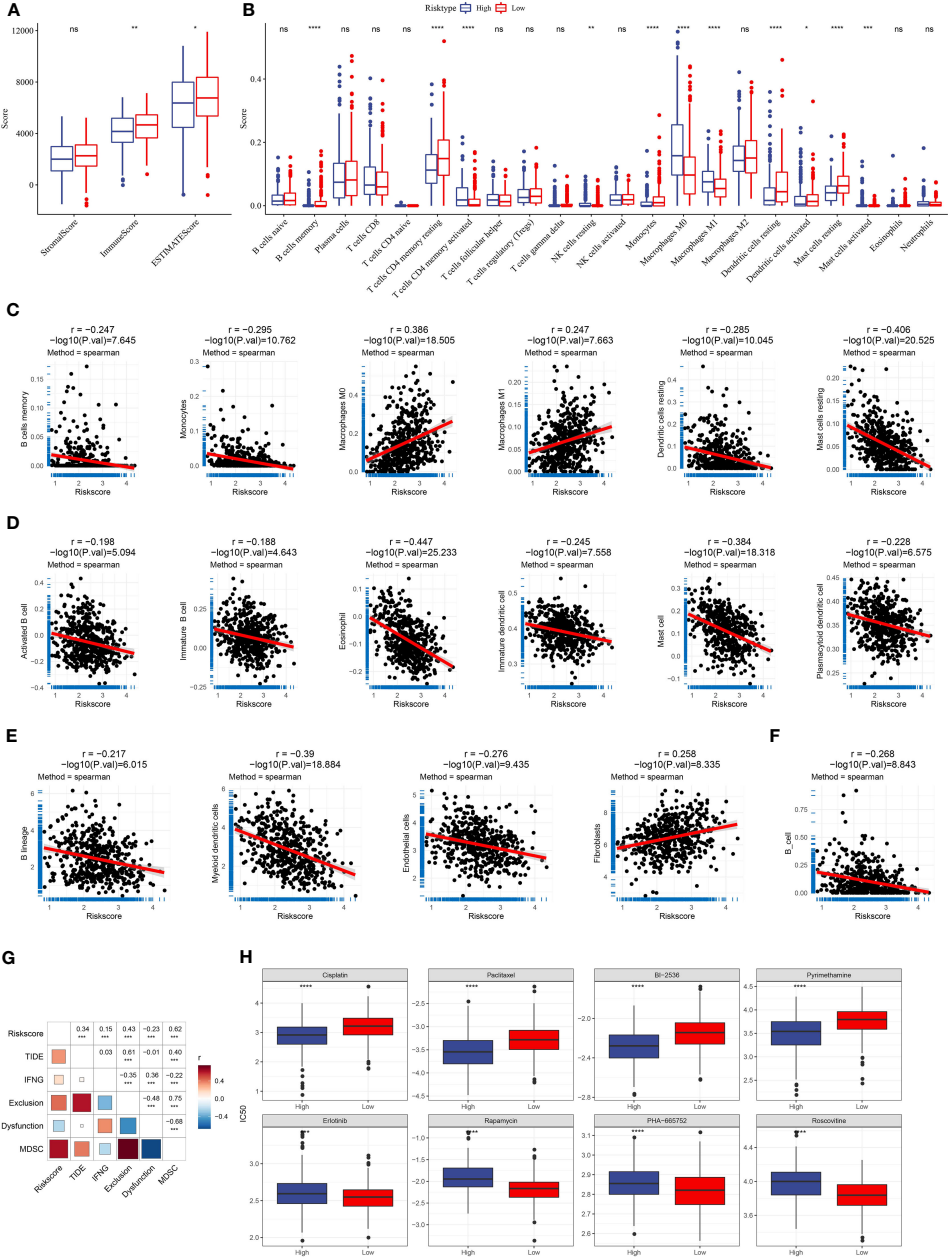
The relation of risk score to immune infiltration and chemotherapeutic response. **(A)** Immune and stromal infiltration of the two risk groups were calculated by the ESTIMATE algorithm. **(B)** The estimated enrichment of 22 immune cells in the two risk groups was calculated by CIBERSORT. **(C–F)** Spearman correlation analysis of risk score with the enrichment of different immune cells and stromal cells was calculated by different tools [CIBERSORT **(C)**, ssGSEA **(D)**, MCP-counter **(E)**, and TIMER **(F)**]. Significant correlation pairs were presented. **(G)** Correlation heatmap of risk score with TIDE, IFN-γ, T cell exclusion, T cell dysfunction, and MDSC. **(H)** The estimated IC50 of eight chemotherapeutic drugs in the high- and low-risk groups. *p < 0.05, **p < 0.01, ***p < 0.001, ****p < 0.0001; ns, no significant.

The immune microenvironment in high-risk and low-risk groups was assessed with different tools and methodologies. ESTIMATE demonstrated that the low-risk group had obviously higher immune scores and stromal scores than the high-risk group, indicating higher stromal infiltration and immune infiltration in the low-risk group (**Figure 8A**). CIBERSORT algorithm revealed that several immune cells were differentially enriched between the two risk groups, such as dendritic cells, M0 macrophages, mast cells, memory CD4 T cells, and M1 macrophages (**Figure 8B**). Using the marker genes of five immune cells obtained by scRNA analysis, the ssGSEA finding indicated that the low-risk group had a higher immune cell score (**Figure S5**). This demonstrated that the two risk groups had differential immune infiltration. Correlation analysis further supported an association of the risk score with immune cell infiltration (**Figures 8C–F**). For instance, B cell lineage (including memory B cells, monocytes, activated B cells, and immature B cells) was negatively correlated with risk score. M0 macrophages, M1 macrophages, and fibroblasts were positively related to risk scores.

### The potential of the risk score in predicting immunotherapy and chemotherapy

TIDE tool can predict the response to immune checkpoint inhibitors (ICIs). We evaluated the enrichment score of myeloid-derived suppressor cells (MDSCs), T cell dysfunction, and T cell exclusion and calculated the TIDE score. We observed that risk score was positively related to TIDE score (P < 0.001, R = 0.34, **Figure 8G**), suggesting that patients with high-risk scores were more vulnerable in the response to ICIs than those with low-risk scores. Notably, the risk score was also positively associated with T cell exclusion (P < 0.001, R = 0.43) and MDSC (P < 0.001, R = 0.62), which may contribute to a negative response to ICIs. The correlation analysis between RiskScore and immune checkpoint genes showed that RiskScore was positively correlated with immune checkpoint genes, indicating that patients in the high-risk group were more suitable for ICB therapy (**Figure S6**). In addition, we used a pRRophetic package to predict the response to chemotherapeutic drugs. The two risk groups had distinct sensitivity to different chemotherapeutic drugs (**Figure 8H**). The high-risk group was more sensitive to Cisplatin, Paclitaxel, BI-2536, and Pyrimethamine, the while low-risk group was more sensitive to Erlotinib, Rapamycin, PHA-665752, and Roscovitine.

### The performance of the seven-gene risk model in immunotherapy-treated patients

**Figure 9:**
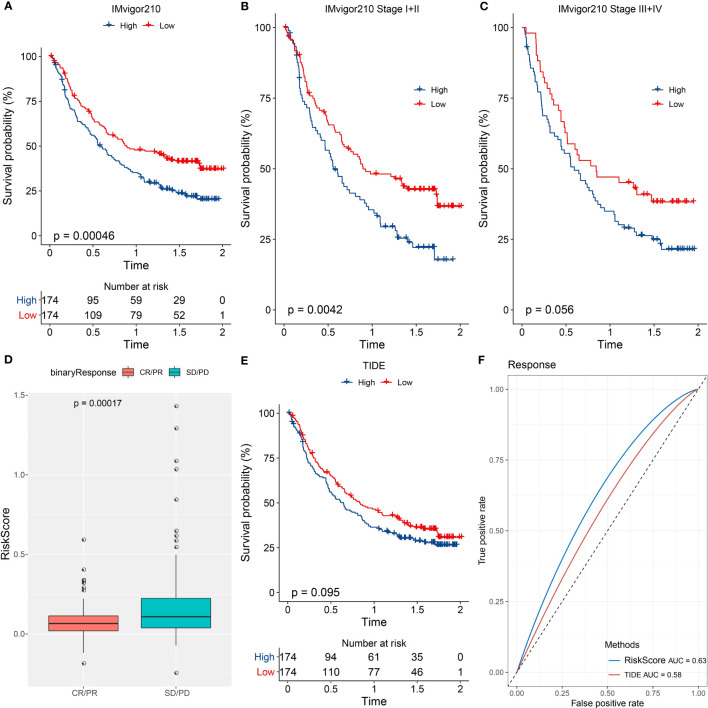
The performance of the risk model in immunotherapy-treated patients. **(A–C)** Kaplan-Meier survival analysis of the two risk groups in all patients **(A)**, patients of early stages (I+II) **(B)**, and patients of late stages (III+IV) **(C)** in the IMvigor210 dataset. **(D)** Comparison of the risk score in CR/PR and SD/PD groups. **(E)** Kaplan-Meier survival analysis of the high- and low-TIDE score groups. **(F)** ROC curve of TIDE score and risk score in the response to immunotherapy.

We further examined the performance of the risk model in the patients administrated by PD-1 inhibitors (IMvigor210 dataset). The same procedure was performed to divide patients into high- and low-risk groups. Survival analysis manifested that the low-risk group had markedly longer overall survival than the high-risk group (**Figure 9A**). Notably, the grouping efficiency was higher in the patients with early stages (I+II) than those with late stages (III+IV) (**Figures 9B, C**). In the IMvigor210 dataset, patients were divided into four groups of different responses to PD-1 inhibitors including CR, PR, SD, and PD where CR and PR indicated positive responses to immunotherapy. We compared the risk score between CR/PR and SD/PD groups. Unsurprisingly, the CR/PR group had a significantly lower risk score than the SD/PD group (P = 0.00017, **Figure 9D**). TIDE score was unable to significantly distinguish between high-risk and low-risk groups (P = 0.095, **Figure 9E**). The AUC of the risk score reached 0.63 while it was 0.58 in the TIDE score (**Figure 9F**).

### Seven key genes were validated by RT-qPCR and Western blot assay

**Figure 10:**
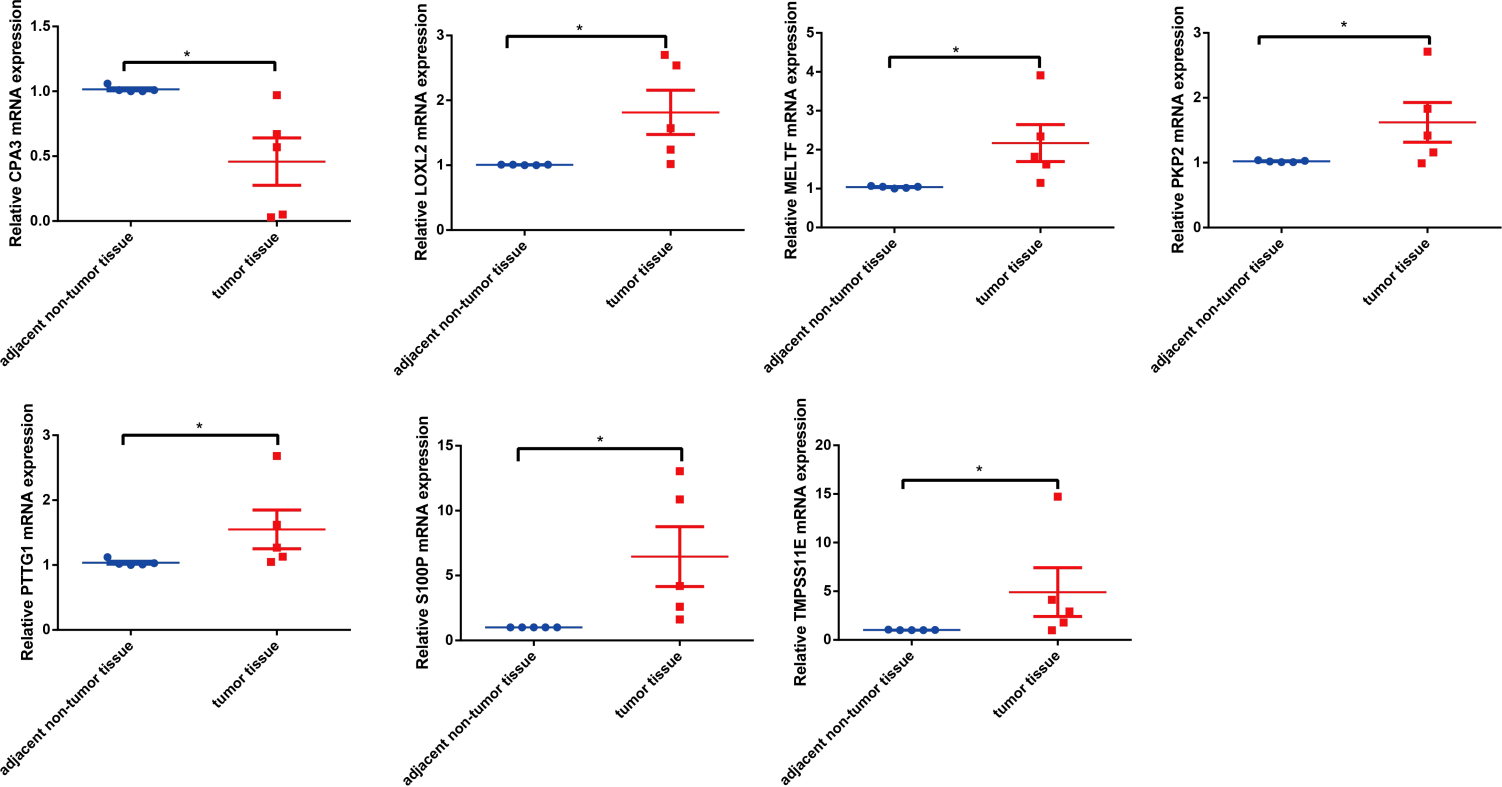
The mRNA levels of seven genes were determined by RT-qPCR. *p < 0.05.

**Figure 11:**
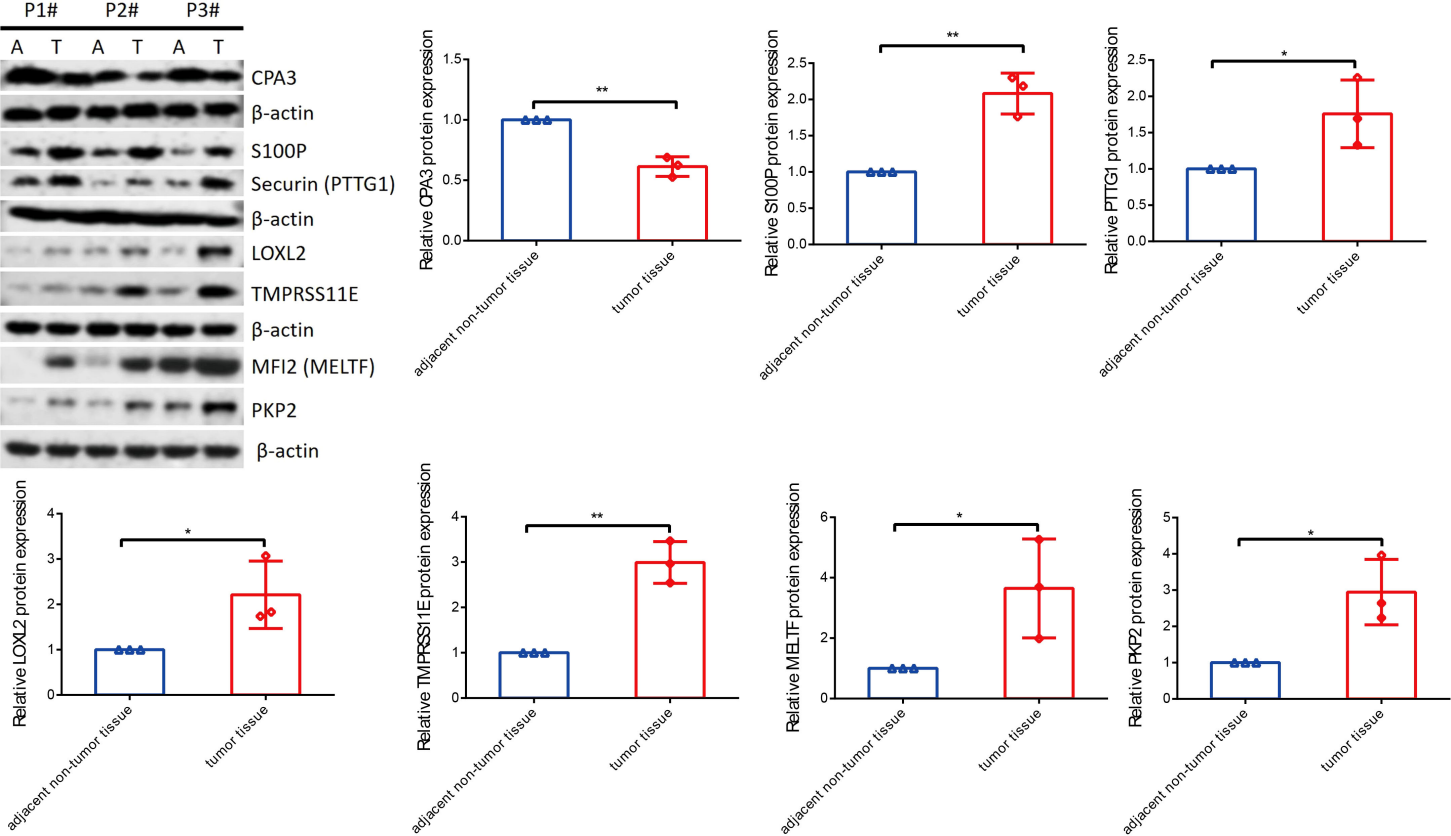
The protein levels of seven genes were detected by Western blot. *p < 0.05, **p < 0.01.

To know the expression profile of CPA3, S100P, PTTG1, LOXL2, MELTF, PKP2, and TMPRSS11E, we used RT-qPCR and Western blot assay to explore the expression in clinical patient tissues. As shown in **Figure 10**, the mRNA level of CAP3 was downregulated, while S100P, PTTG1, LOXL2, MELTF, PKP2, and TMPRSS11E mRNA levels were upregulated in LUDA tissues in comparison to paracarcinoma. Surprisingly, the protein levels of seven genes had a similar trend in tumor tissues (**Figure 11**).

## Discussion

In this study, we utilized scRNA-seq data and bulk RNA-seq data to explore prognostic genes associated with LUAD and immunity. We identified three clusters (C1, C2, and C3) with distinct clinical and molecular features. Through differential analysis between different clusters, we detected a series of DEGs. We further excavated seven key prognostic genes from these DEGs to establish a risk model. The risk model was effective in discriminating between high-risk and low-risk patients, which could provide guidance for clinical treatment.

C1 had the longest overall survival whereas C3 had the worst prognosis. Consistent with clinical characteristics, samples with advanced stages had a higher proportion in C3 compared with the other two clusters. In addition, C3 had the largest number of dead samples. To clarify the molecular mechanisms causing the different prognoses of the three clusters, we analyzed their immune characteristics, biological pathways, and genomic features. C1 had the highest immune infiltration while C3 had the lowest immune infiltration. The immune microenvironment has been demonstrated to be extraordinarily associated with anti-cancer immune response and prognosis (32). Previous research has shown that high immune infiltration is associated with a good prognosis in lung cancer (33, 34), which is consistent with our result. However, C3 showed higher immune infiltration than C2. We further analyzed the enrichment of tumor-related pathways. Not surprisingly, C3 manifested higher enrichment of oncogenic pathways such as VEGF, hypoxia, EGFR, and MAPK than the other two clusters, which were responsible for the worse prognosis of C3. In addition, C3 had high levels of TMB, aneuploidy, homologous recombination defect, fraction altered, and number of segments, suggesting the genomic instability of C3. Genomic instability is one of cancer hallmarks, which is closely associated with cancer development (35, 36). Molecular analysis of the three clusters explained their different prognosis and the reliability of molecular subtyping based on immune cell markers.

Furthermore, we assessed DEGs between different clusters, and a total of 133 DEGs were identified. KEGG analysis revealed that these DEGs were significantly enriched in the cell cycle, implying that the dysregulation of the cell cycle pathway greatly contributed to the different outcomes of the three clusters. Using Lasso and stepAIC algorithms, we confirmed seven key prognostic genes including CPA3, S100P, PTTG1, LOXL2, MELTF, PKP2, and TMPRSS11E to establish a risk model. CPA3 belongs to zinc metalloproteases, which may contribute to the degradation of endogenous proteins and the inactivation of venom-associated peptides. CPA3 was reported as a prognostic biomarker in colon cancer (37), but its role has not been fully investigated in cancers. S100P is an EF-hand calcium-binding protein and its overexpression has been detected in many cancer types (38). The overexpression of S100P promotes cancer progression and metastasis through extracellular signaling via the RAGE receptor or through intracellular interaction with ezrin (38). S100P serves as a potential therapeutic target in cancer treatment. Chien et al. revealed that targeting S100P inhibited cell motility and migration in invasive non-small cell lung cancer (NSCLC) cells (39). PTTG1 is also detected to be overexpressed in multiple cancers such as breast cancer, lung cancer, and gastric cancer (40). PTTG1 is involved in regulating the transcription of basic fibroblast growth factor, activating c-Myc, and promoting apoptosis of specific cell types (41, 42). Knockdown of PTTG1 strengthens the anti-cancer immune response in LUAD (43), implying PTTG1 is a potential target. LOXL2 belongs to the lysyl oxidase (LOX) family and increasing evidence has shown its role in cancer cell invasion and metastasis (44). High expression of LOXL2 is associated with poor prognosis in NSCLC patients (45). Melanotransferrin (MELTF) is identified as a serological biomarker in gastric cancer (46), colorectal cancer (47), and lung cancer (48). Plakophilins 2 (PKP2) was found to promote LUAD cell proliferation and migration through epithelial-mesenchymal transition and EGFR signaling (49, 50). Few papers have discussed TMPRSS11E’s function in malignancies.

In the TCGA and GSE31210 datasets, the seven-gene risk model performed satisfactorily in predicting the prognosis of LUAD. Compared to patients with a potentially high risk, more immunological infiltration and a better prognosis were found in the low-risk group. The risk score and TIDE score were positively correlated, showing that high-risk groups had a higher likelihood of avoiding immunotherapy. In response to chemotherapy, the two risk groups also showed different IC50. The high-risk group was more sensitive to Cisplatin, Paclitaxel, BI-2536, and Pyrimethamine, while the low-risk group was more sensitive to Erlotinib, Rapamycin, PHA-665752, and Roscovitine. These results supported that the risk model may provide a direction for treating LUAD patients with immunotherapy and chemotherapeutic drugs.

## Conclusions

In conclusion, this study integrated the analysis of scRNA-seq data and bulk RNA-seq data to identify molecular subtypes for LUAD patients. We focused on immune cell marker genes and confirmed three clusters with different prognoses, clinical features, immune infiltration, and genomic features. Importantly, we constructed a seven-gene risk model which was reliable and effective to predict prognosis and guide personalized therapies for LUAD patients.

## Data availability statement

The original contributions presented in the study are included in the article/**Supplementary Material**, further inquiries can be directed to the corresponding author/s.

## Ethics statement

The studies involving human participants were reviewed and approved by Ethics Committee of Ningbo No.2 Hospital. The patients/participants provided their written informed consent to participate in this study.

## Author contributions

The study was designed by JS and JJ. GZ participated in the literature review. JJ performed the data analysis and interpretation. The original draft of the manuscript was done by GZ. JS reviewed and edited the manuscript. The manuscript was reviewed and approved by all authors.
