# Supplementary figures and images for: Identification of novel gene signature for lung adenocarcinoma by machine learning to predict immunotherapy and prognosis
Source: Front Immunol. 2023 Jul 31;14:1177847. doi: 10.3389/fimmu.2023.1177847 (PMC10424935; doi:10.3389/fimmu.2023.1177847)

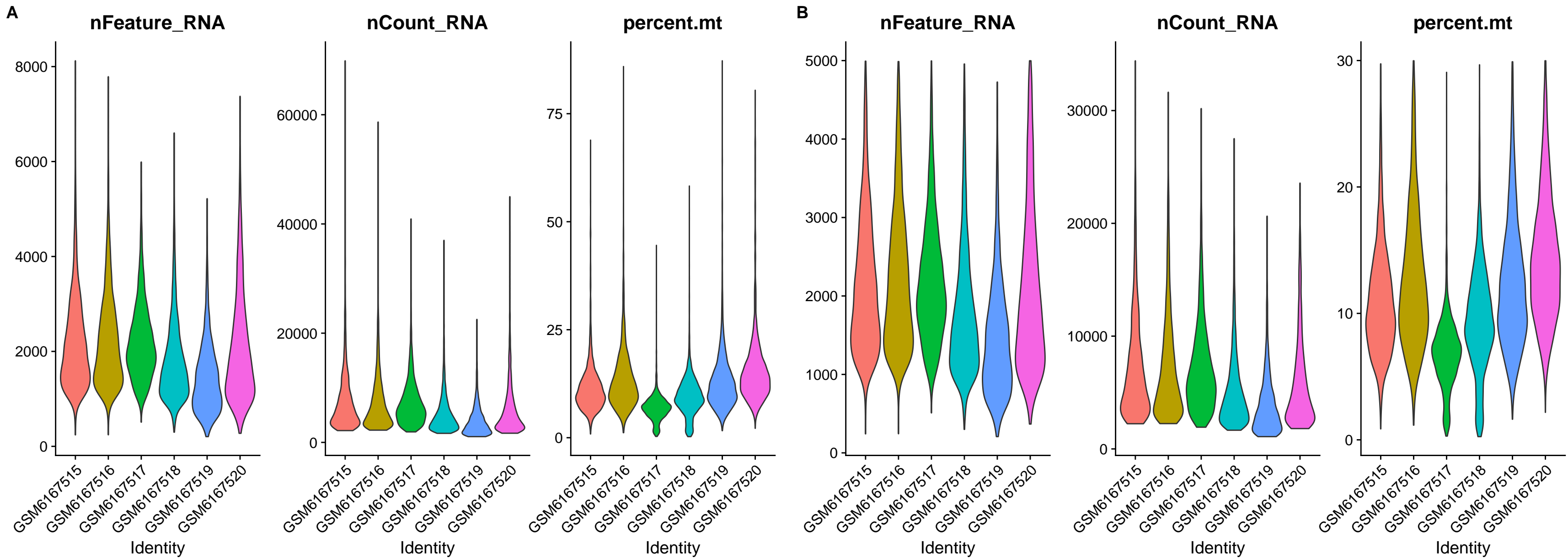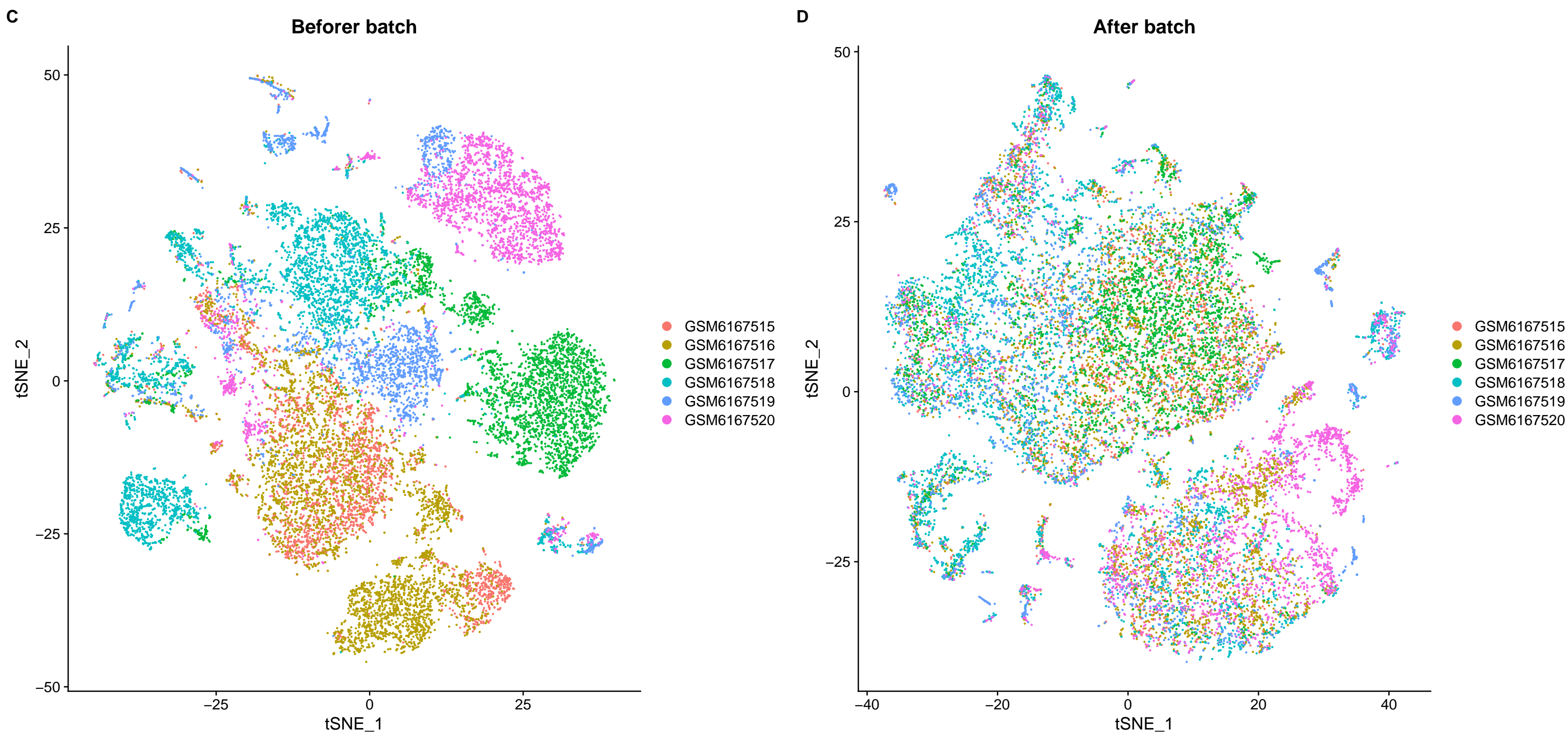

Supplement: Supplementary file 1 [file DataSheet_1.pdf]

A

consensus CDF

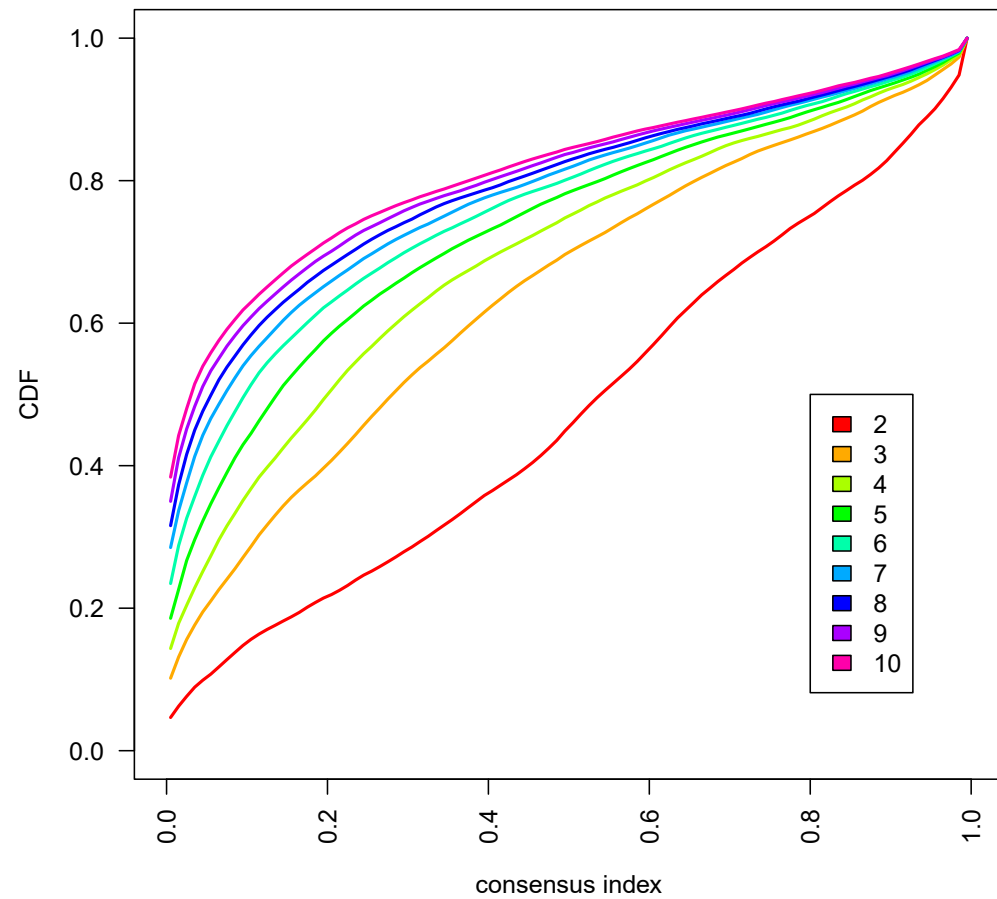

B

Delta area

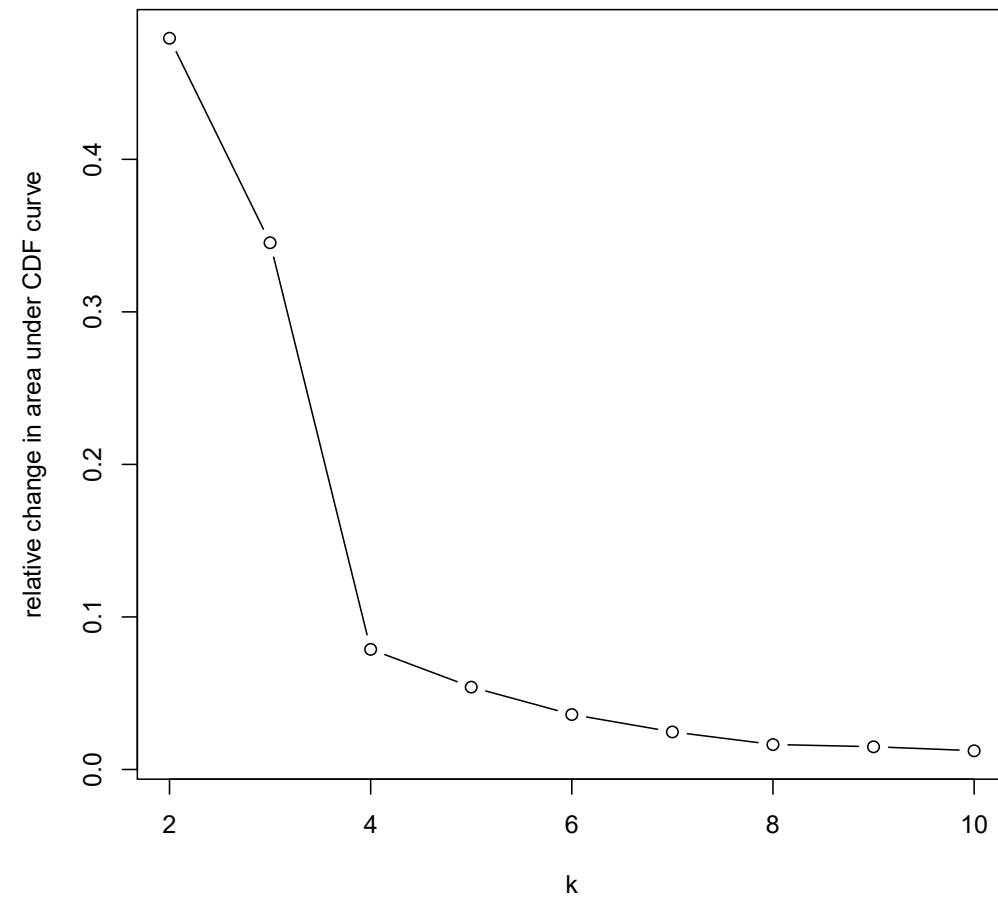

C

consensus matrix  $k=3$ 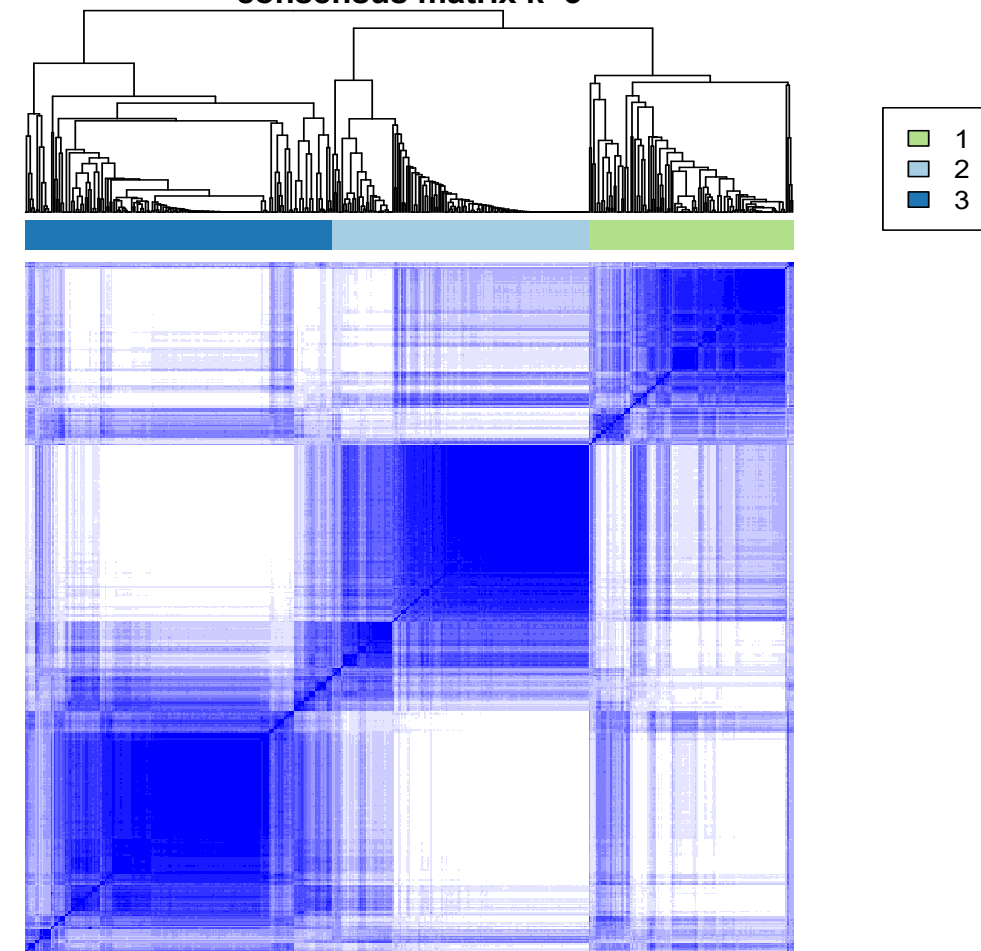

Supplement: Supplementary file 2 [file DataSheet_2.pdf]

**A** C1 vs Other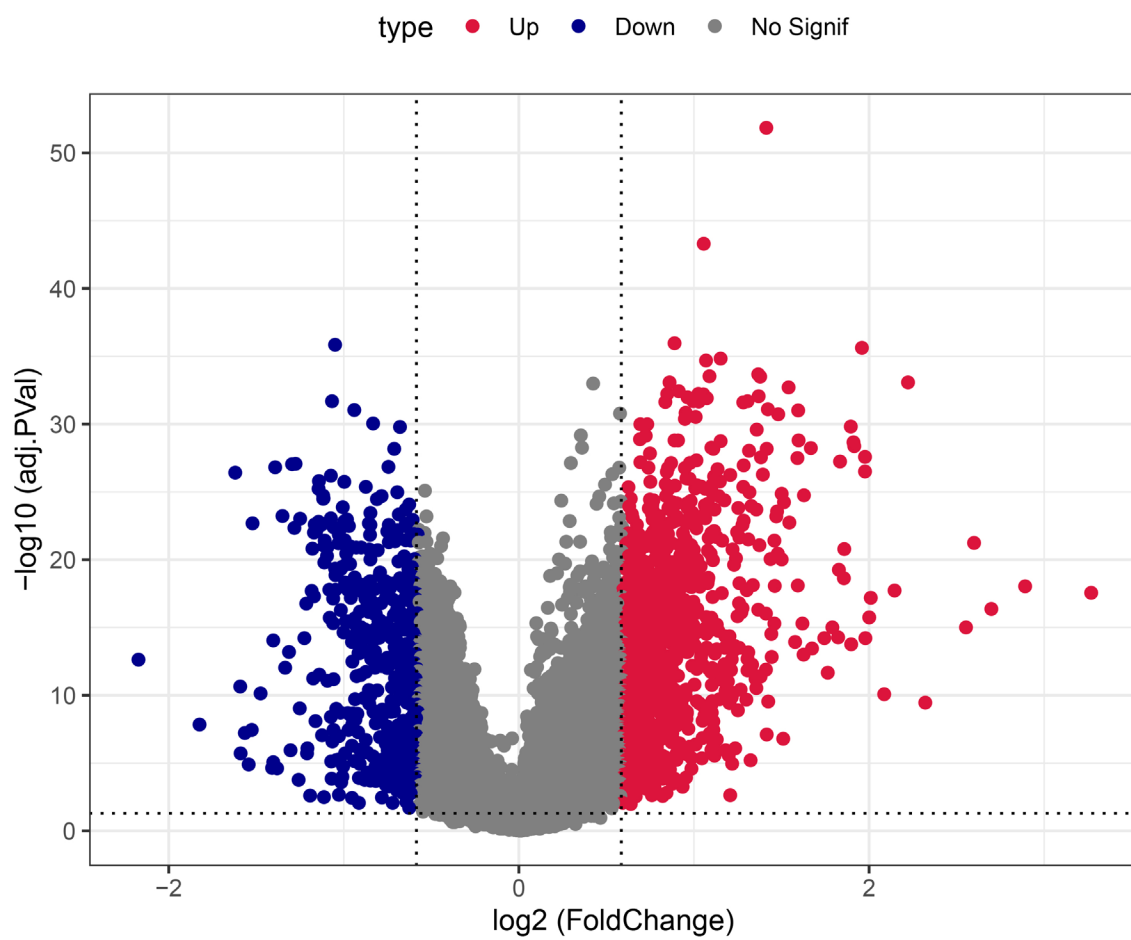**B** C2 vs Other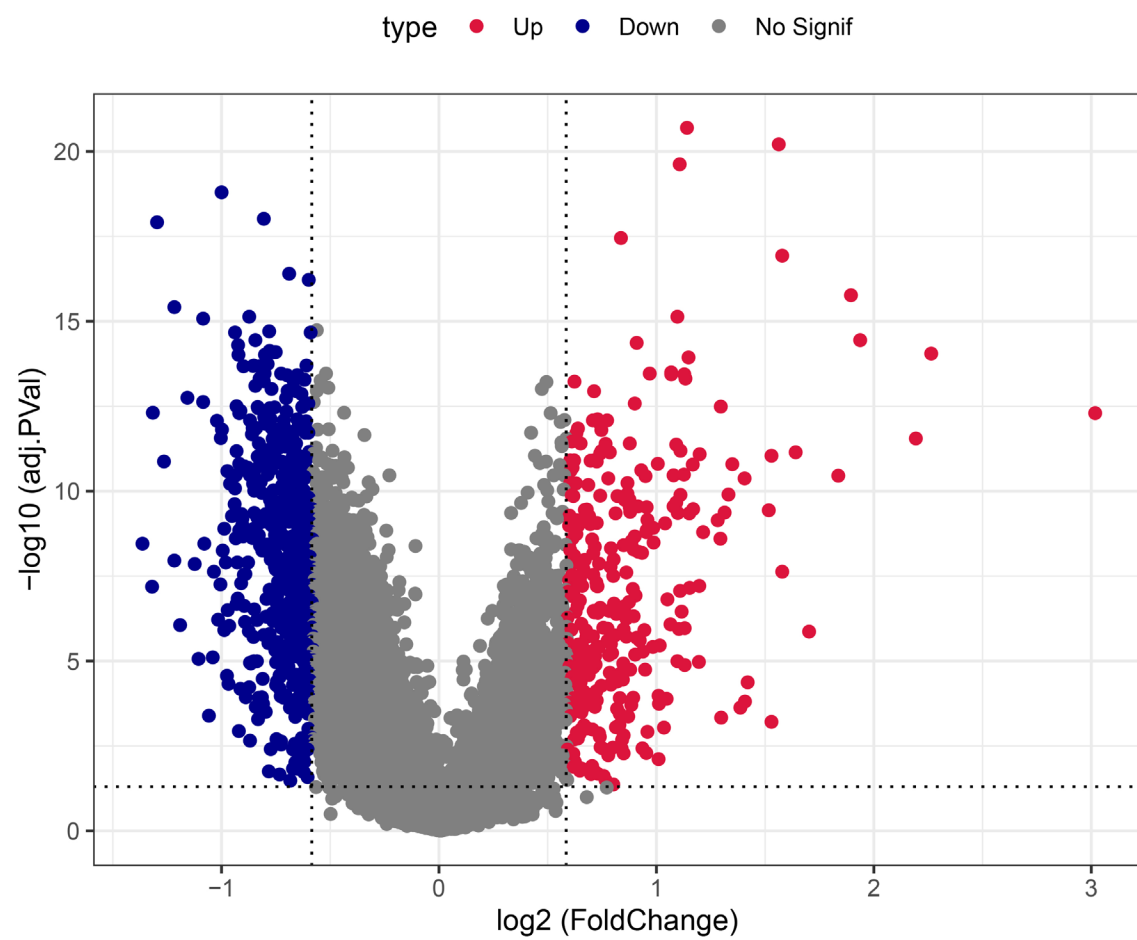**C** C3 vs Other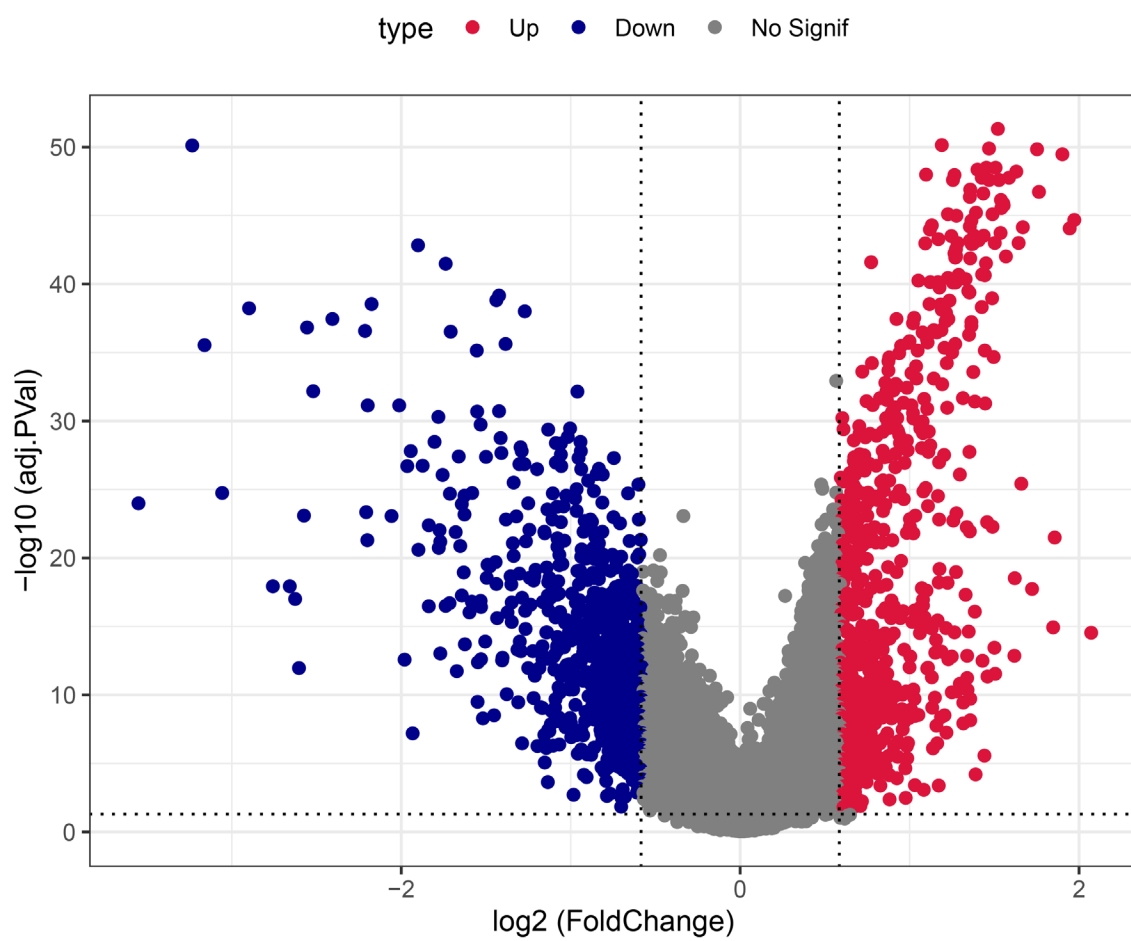**D**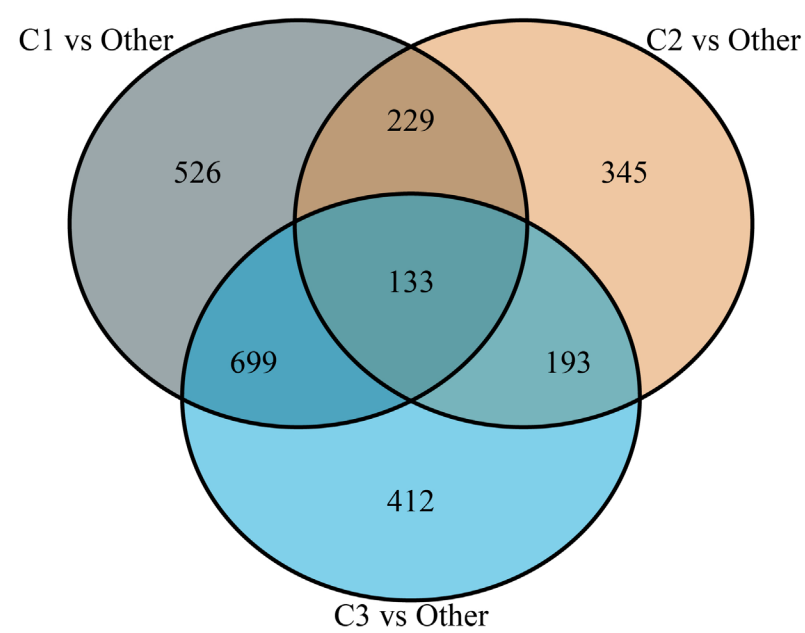**E**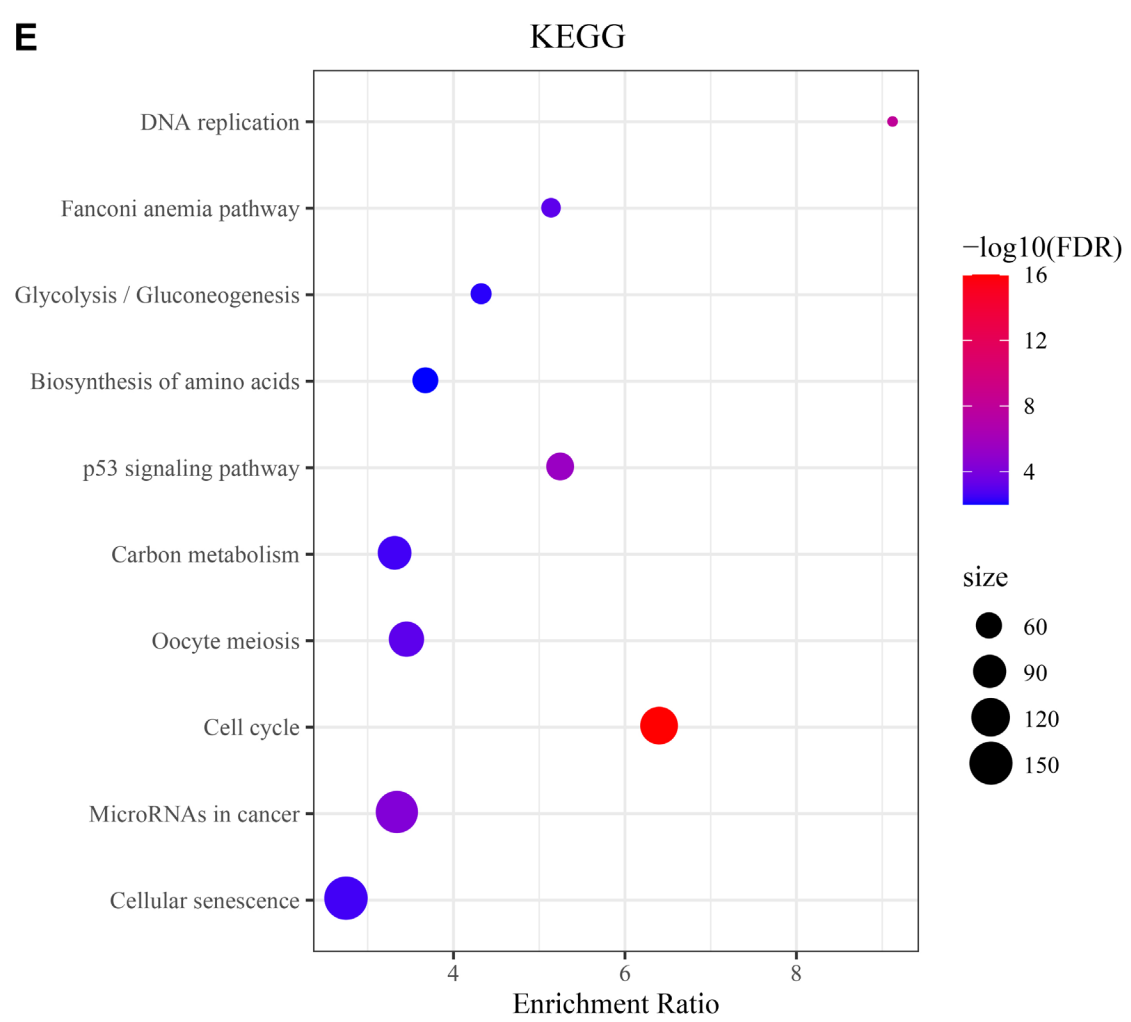**F**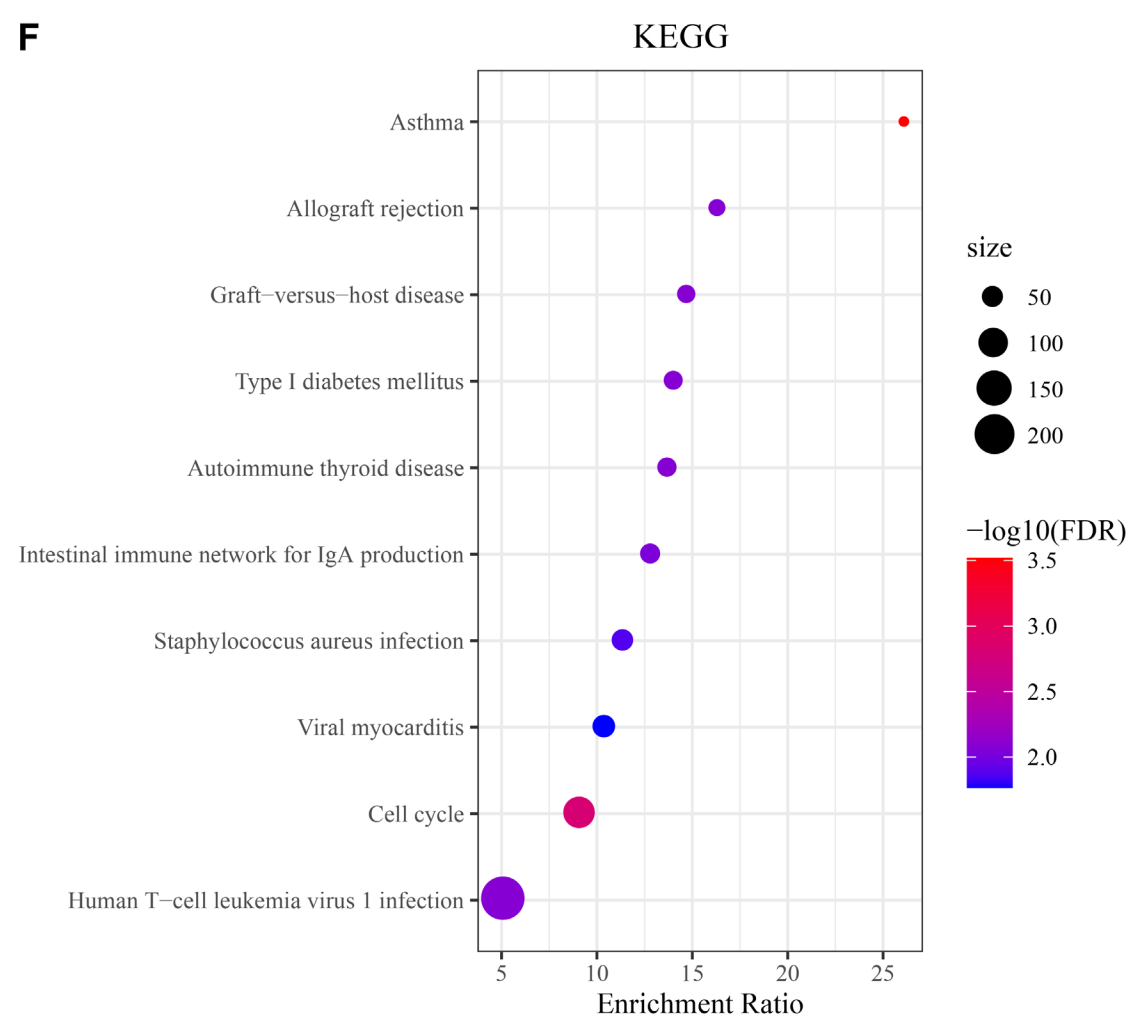

Supplement: Supplementary file 3 [file DataSheet_3.pdf]

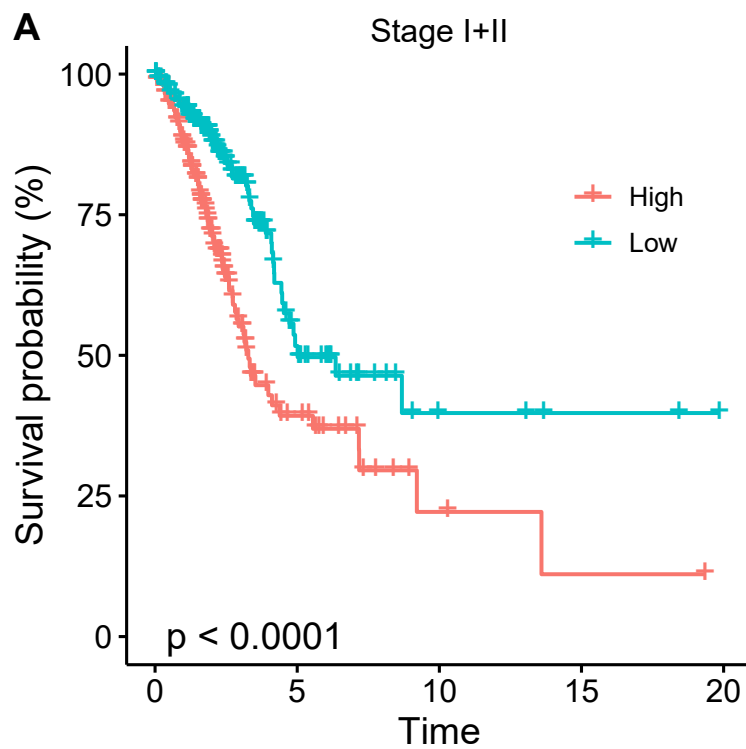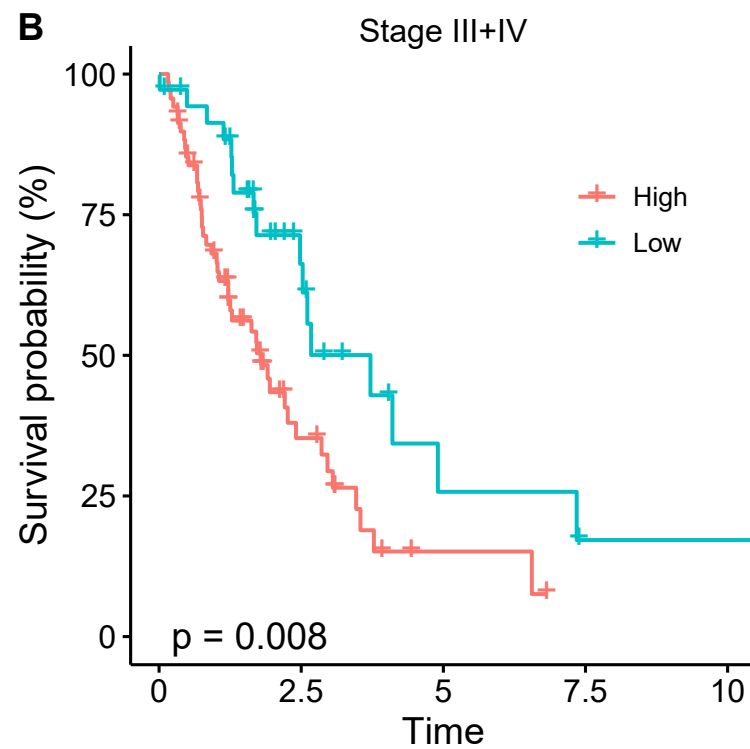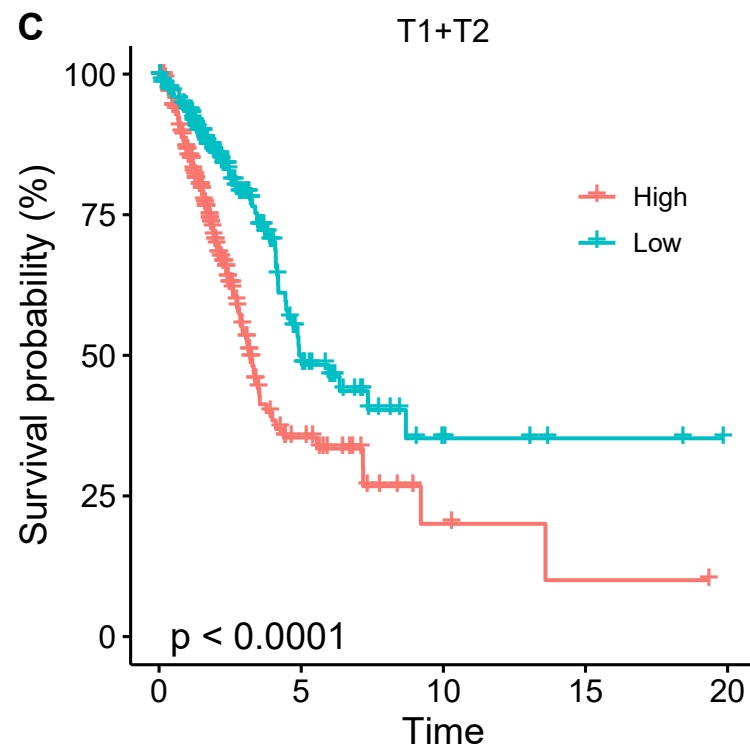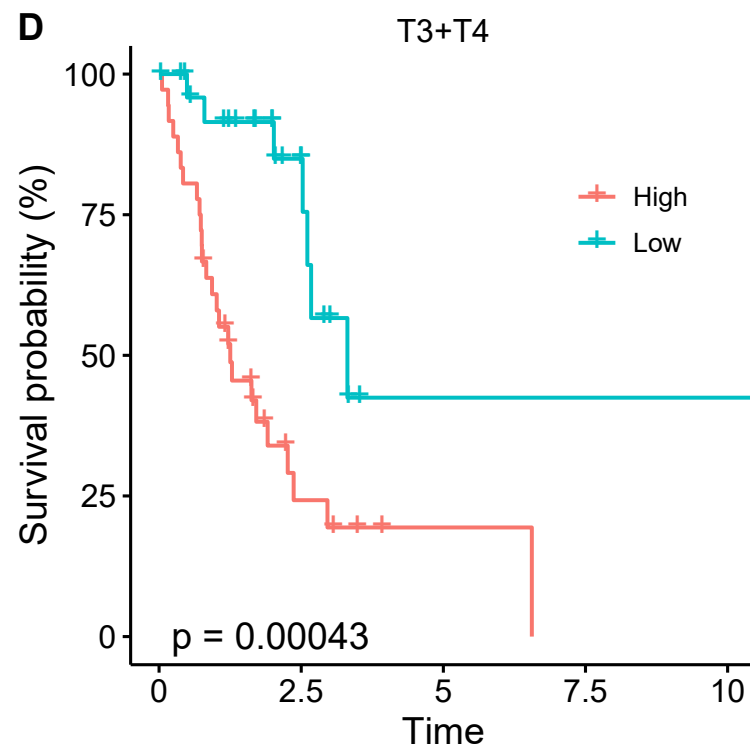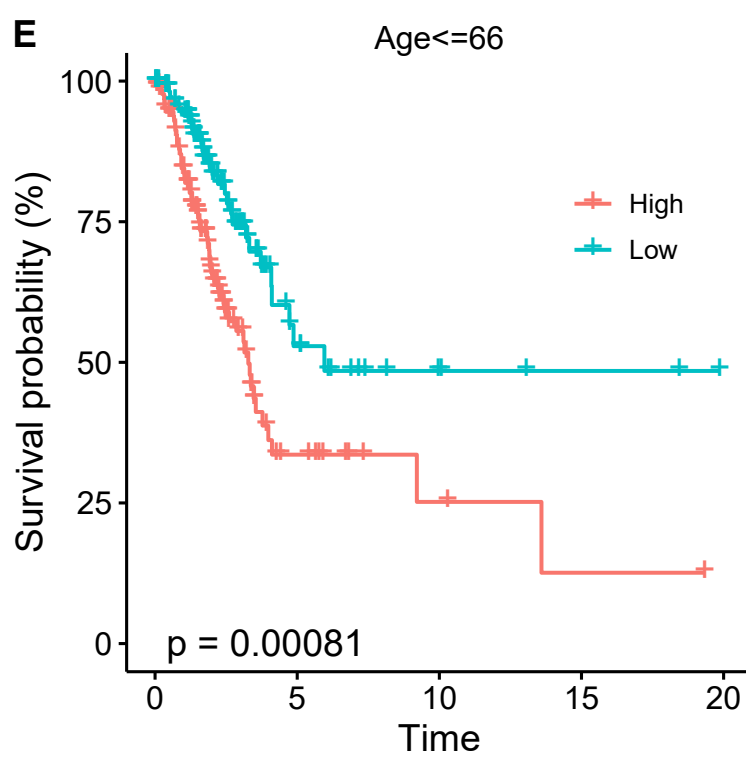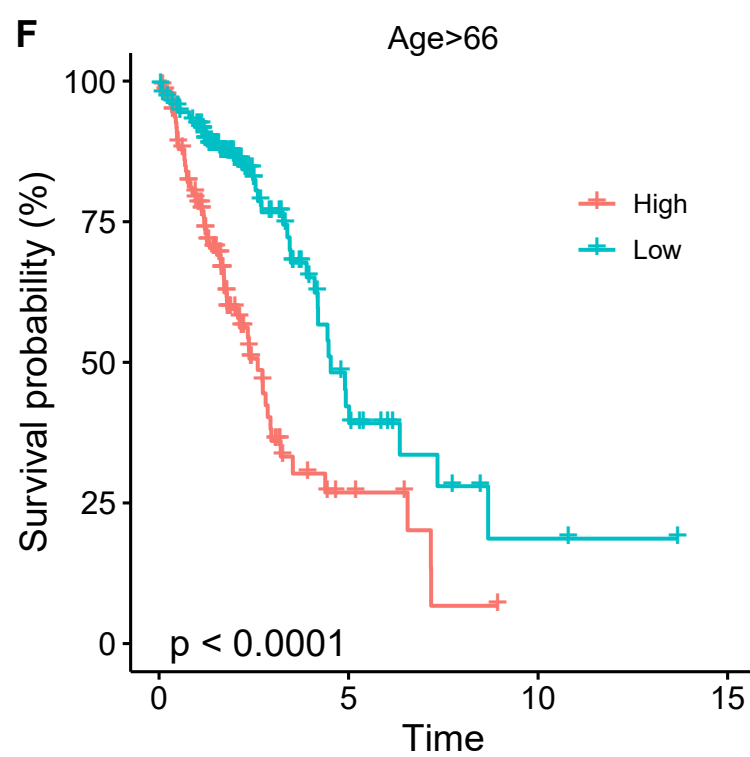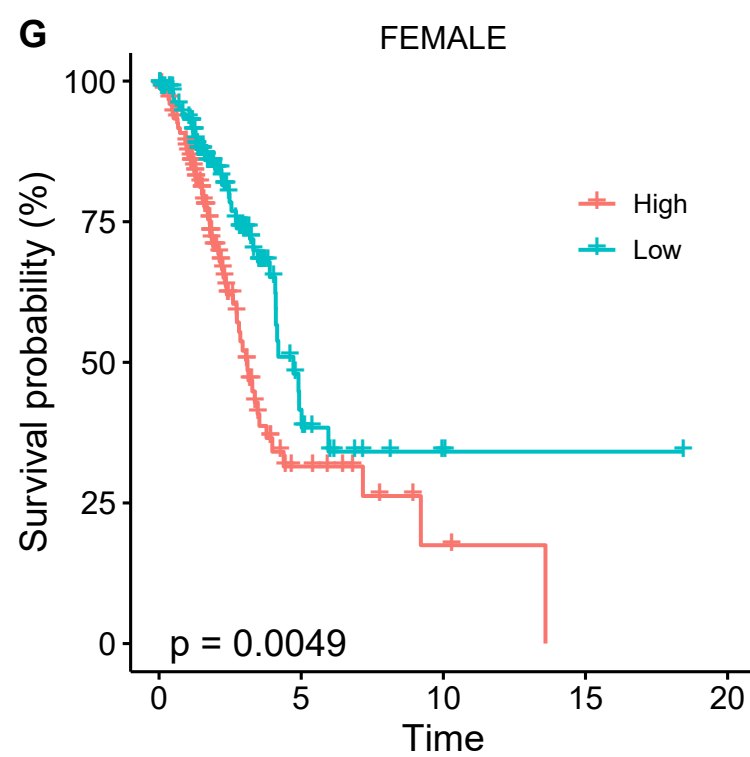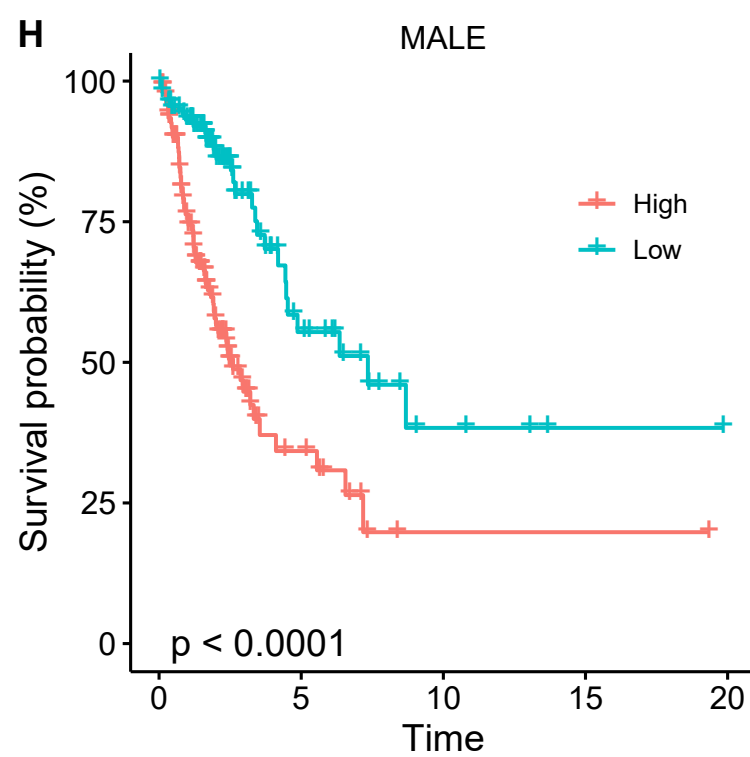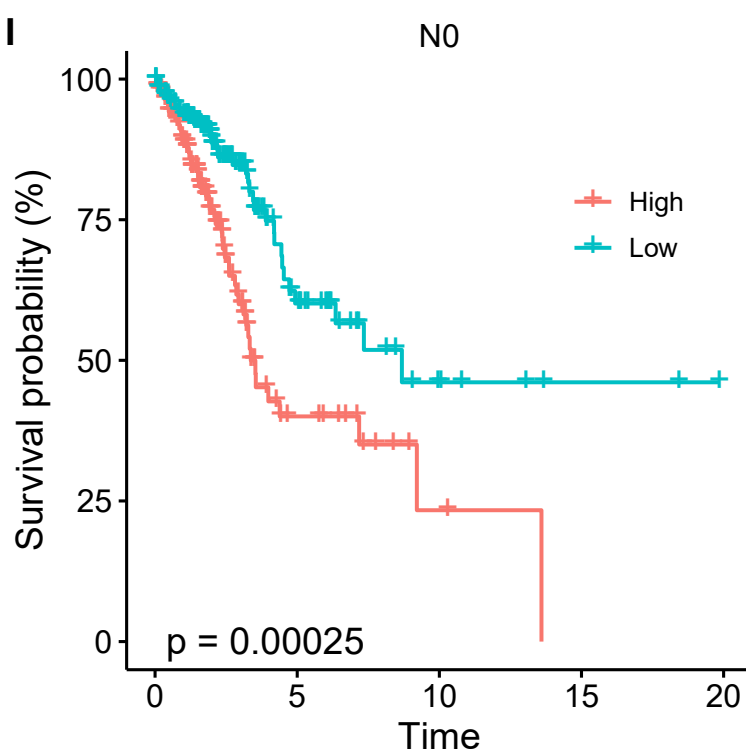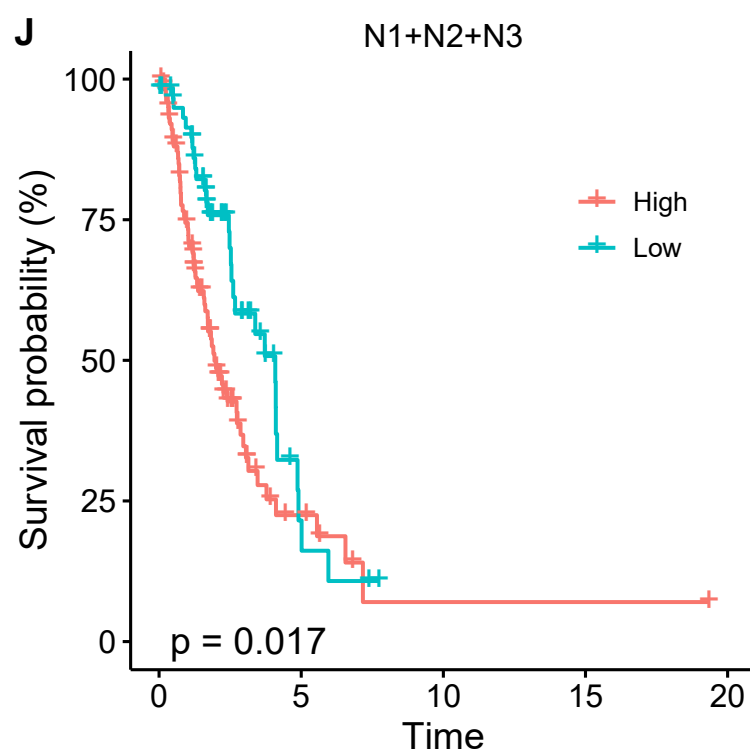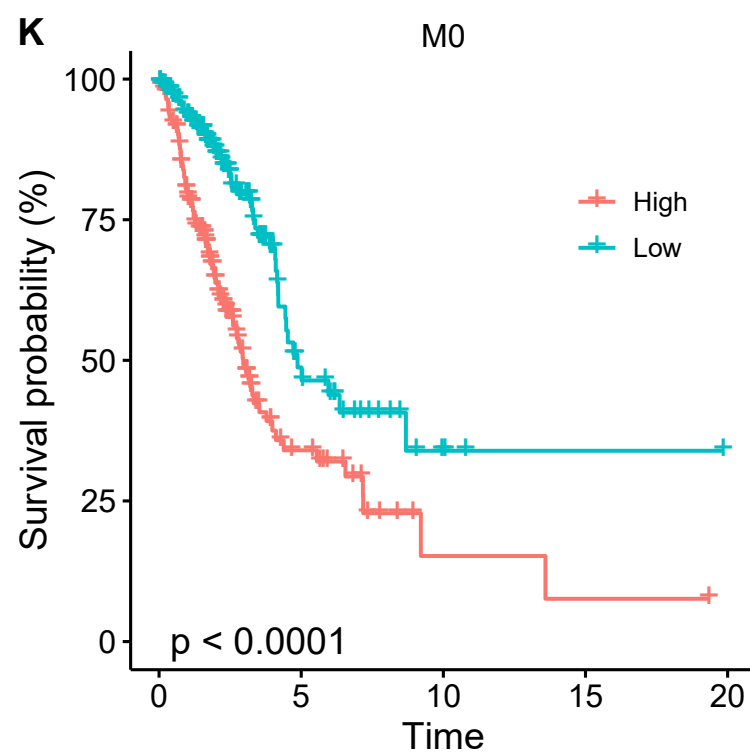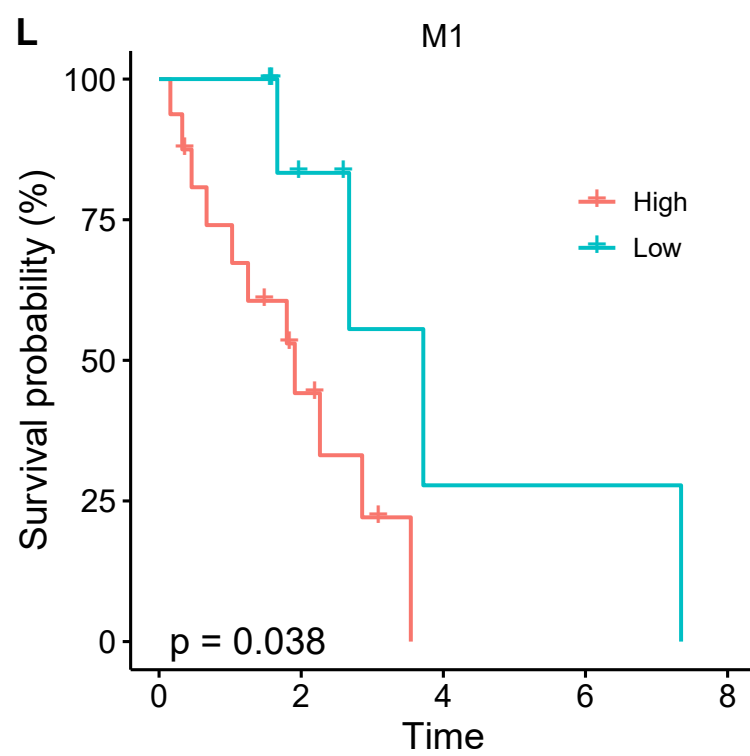

Supplement: Supplementary file 4 [file DataSheet_4.pdf]

category High Low

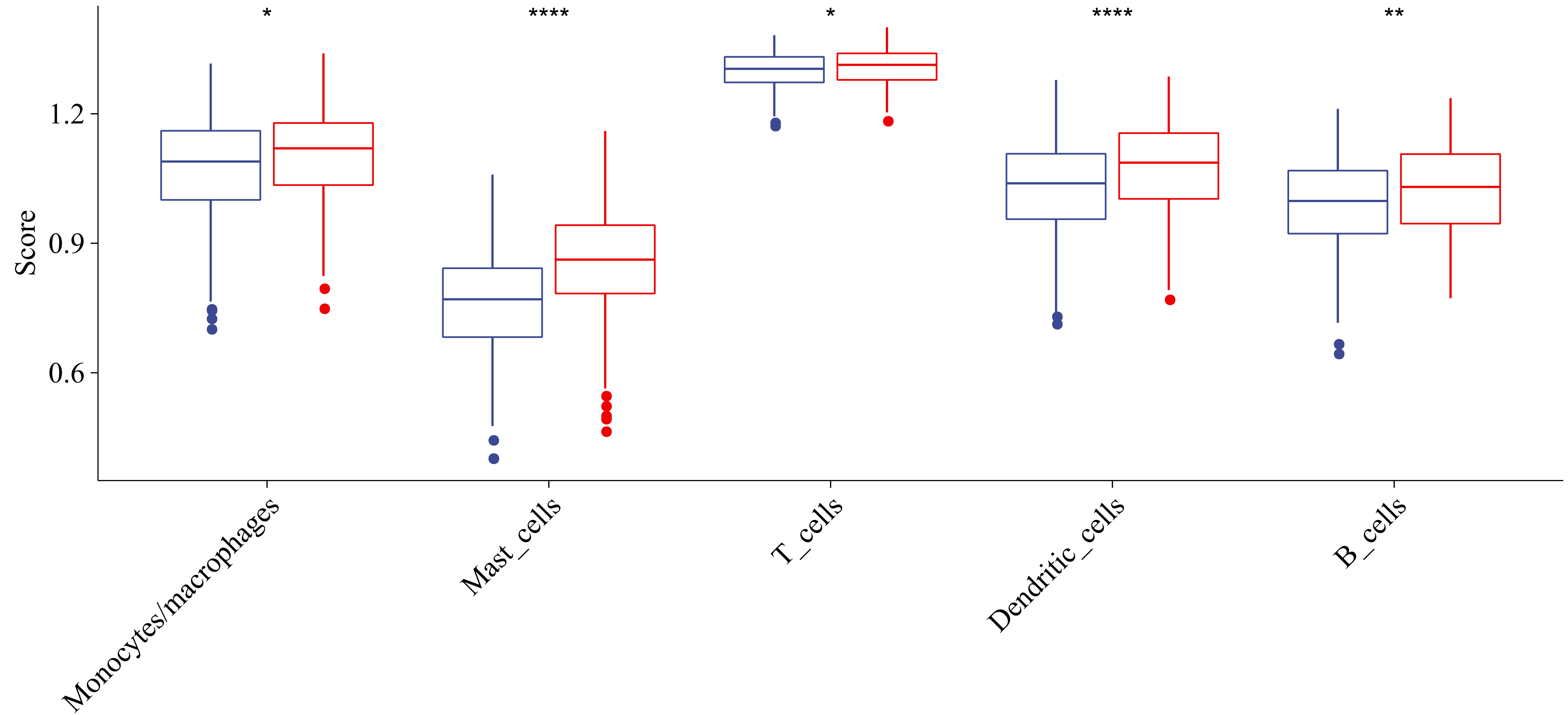

Supplement: Supplementary file 5 [file DataSheet_5.pdf]

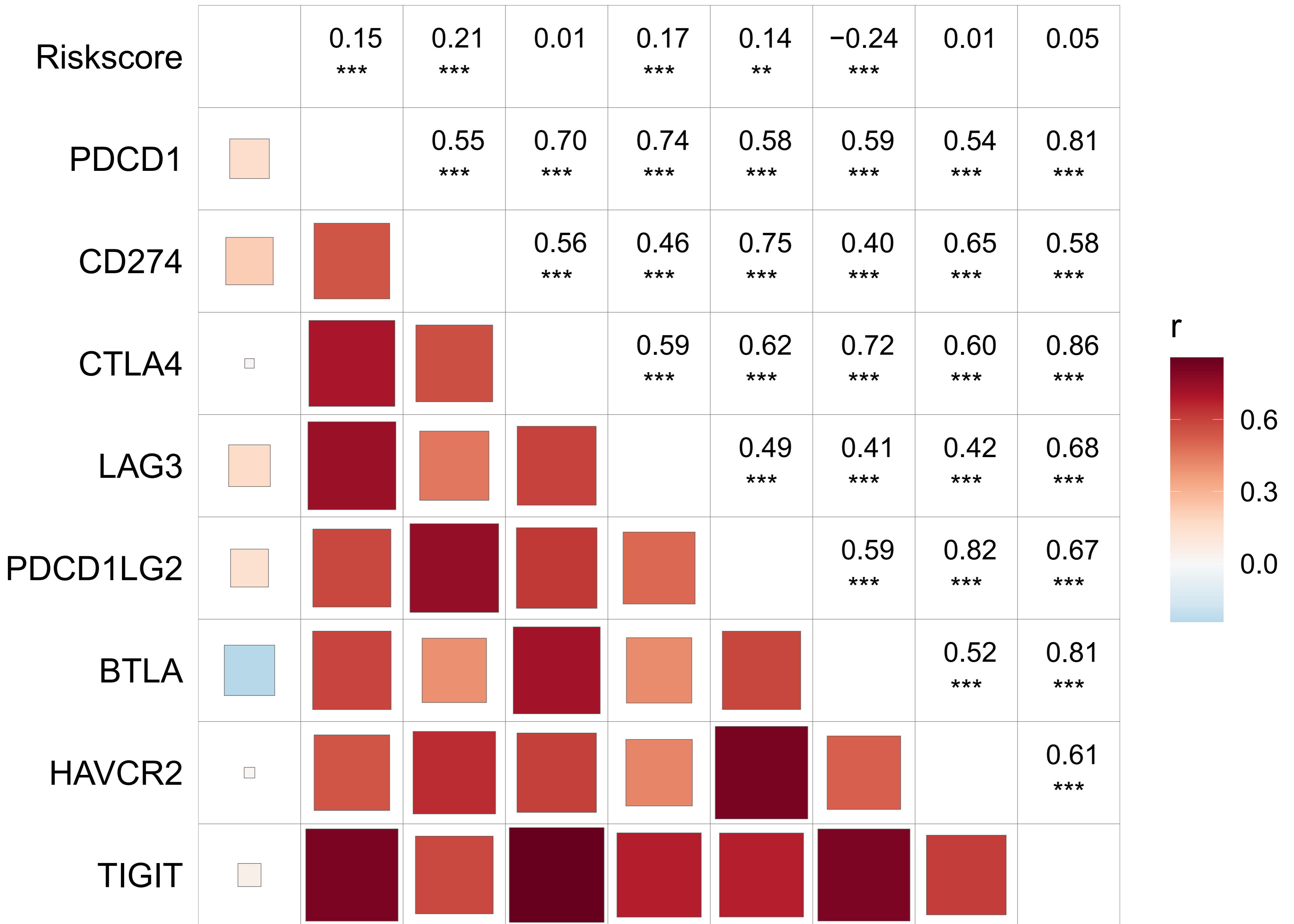

Supplement: Supplementary file 6 [file DataSheet_6.pdf]
